# Rapid mechanochemical encapsulation of biocatalysts into robust metal–organic frameworks

**DOI:** 10.1038/s41467-019-12966-0

**Published:** 2019-11-01

**Authors:** Tz-Han Wei, Shi-Hong Wu, Yi-Da Huang, Wei-Shang Lo, Benjamin P. Williams, Sheng-Yu Chen, Hsun-Chih Yang, Yu-Shen Hsu, Zih-Yin Lin, Xin-Hua Chen, Pei-En Kuo, Lien-Yang Chou, Chia-Kuang Tsung, Fa-Kuen Shieh

**Affiliations:** 1 School of Physical Science and Technology, ShanghaiTech University, Shanghai, 201210 China; 2 Department of Chemistry, National Central University, Taoyuan, 32001 Taiwan; 3 Department of Chemistry, Merkert Chemistry Center, Boston College, Chestnut Hill, MA 02467 USA

**Keywords:** Biocatalysis, Enzymes, Metal-organic frameworks

## Abstract

Metal–organic frameworks (MOFs) have recently garnered consideration as an attractive solid substrate because the highly tunable MOF framework can not only serve as an inert host but also enhance the selectivity, stability, and/or activity of the enzymes. Herein, we demonstrate the advantages of using a mechanochemical strategy to encapsulate enzymes into robust MOFs. A range of enzymes, namely β-glucosidase, invertase, β-galactosidase, and catalase, are encapsulated in ZIF-8, UiO-66-NH_2_, or Zn-MOF-74 via a ball milling process. The solid-state mechanochemical strategy is rapid and minimizes the use of organic solvents and strong acids during synthesis, allowing the encapsulation of enzymes into three prototypical robust MOFs while maintaining enzymatic biological activity. The activity of encapsulated enzyme is demonstrated and shows increased resistance to proteases, even under acidic conditions. This work represents a step toward the creation of a suite of biomolecule-in-MOF composites for application in a variety of industrial processes.

## Abstract (web summary)

Metal–organic frameworks (MOFs) are attractive for encapsulating enzymes for industrial purposes because they can increase selectivity, stability, and/or activity of the enzymes. Here, the authors developed an economical solid-state mechanochemical method to encapsulate enzymes during MOF synthesis.

## Introduction

Enzymes are natural catalysts with high specificity that underpin life on Earth. For decades, humans have benefited from their use in both scientific research and industrial application^1–3^. When enzymes are employed as industrial catalysts, they are often immobilized on solid supports to enhance their robustness and provide a heterogeneous environment for easy separation^4–7^. Metal–organic frameworks (MOFs) have recently garnered consideration as an attractive solid substrate because the highly tunable MOF framework can not only serve as an inert host but also enhance the selectivity, stability, and/or activity of the enzymes. For selectivity, it has been reported that MOFs can endow enzymes with size selectivity, prohibiting chemicals larger than the designed aperture from reaching the catalyst^8–11^. For stability, it has been reported that the interaction with the MOF framework could provide increased resiliency to the enzymes^12–15^. Recent detailed mechanistic studies have shown, for example, that the spatial confinement provided by a MOF support can prevent an enzyme from unfolding and losing catalytic activity when it is exposed to denaturing conditions^16–25^. For activity, MOF-encapsulated enzymes have, in certain cases, shown an even higher activity than free enzymes owing to the more-efficient delivery of chemicals induced by the hierarchical porous structure^26^. Further, even when serving simply as host, the MOF synthesis process has allowed multiple different enzymes to be introduced into one MOF crystal to run a cascade catalytic reaction^27^. Overall, recent works indicate that enzymes encapsulated in MOFs represent promising catalysts; as such, a synthetic route applicable to a wider range of enzymes and MOFs is desired.

Mechanochemical processes, such as ball milling, have been shown to be an environment-friendly alternative to traditional solution-based processes and can be scaled to industrial levels^28–31^. They have already been used for the production of a variety of MOFs with a high production rate (kilograms per hour)^32,33^. Mechanochemically, encapsulating enzymes during the MOF synthesis, then, seems like a natural and green next step toward synthesizing supported enzymatic catalysts. Generally, introducing enzymes during synthesis, instead of impregnating enzymes into the already-synthesized framework, is referred as de novo or biomimetic mineralization encapsulation^15,16,24^. The advantages this strategy has over solution-based synthesis have been demonstrated, but only for ZIF-type MOFs, i.e., ZIF-90 or ZIF-8^34^, because the synthetic conditions required for other classes of MOFs such as UiO-66-NH_2_^35^ and Zn-MOF-74^36–38^ are too harsh, requiring organic solvents and far-from-biological pH levels, for the encapsulated enzymes to retain their activity^39^. The mechanochemical process offers an opportunity to avoid this problem because it only uses a trace amount of solvent and enzymes are generally more stable in powder than in solution^40^. Also, the rapid reaction time minimizes enzyme exposure to reaction conditions, limiting the potential detrimental effects of chemicals and reduction of enzymatic activity.

**Fig. 1:**
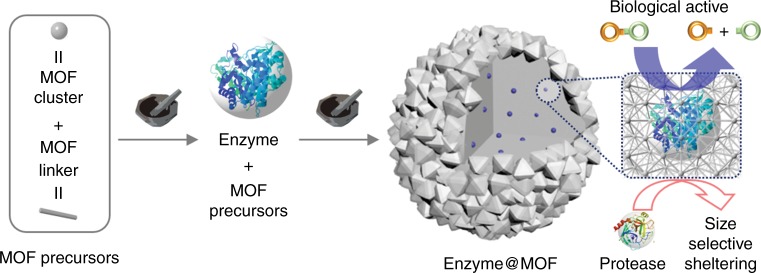
Schematic illustration of the mechanochemical method. The obtained biocomposites via the two-step approach for embedding glycosidases into MOF is shown, illustrating the biological activity and protective effect

Here, we introduce a solid-state mechanochemical strategy to encapsulate enzymes during MOF synthesis. To this end, we perform a proof-of-concept synthesis of MOF-encapsulated enzymes through a solid-state mechanochemical strategy via ball milling (Fig. 1). Three well-studied MOFs with different crystal structures and chemical compositions, UiO-66-NH_2_, ZIF-8, and Zn-MOF-74 are used. It is worth noting that enzyme encapsulation in robust MOFs such as UiO-66 has never been demonstrated owing to the harsh solution-based synthetic conditions, though they are ideal for catalysis because of their high chemical stability relative to other MOFs.

## Results

### Mechanochemically encapsulating

**Fig. 2:**
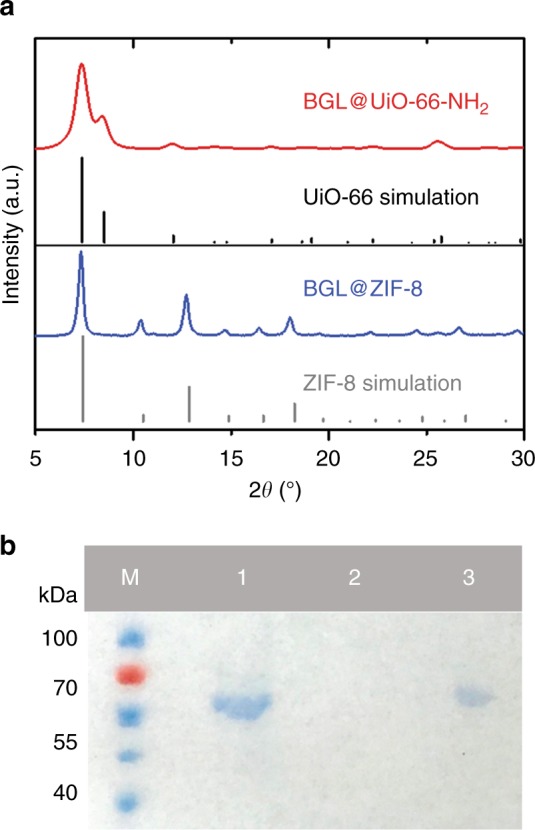
Characteristics of BGL@ZIF-8 and BGL@UiO-66-NH_2_. **a** PXRD patterns of BGL@UiO-66-NH_2_ (two-step approach) and BGL@ZIF-8 and simulations of UiO-66 and ZIF-8. **b** SDS–PAGE gel (M: protein marker, lane 1: free BGL, lane 2: washed BGL-on-UiO-66-NH_2_, and lane 3: BGL@UiO-66-NH_2_). Source data are provided as a Source Data file

As an initial study, we encapsulated β-glucosidase (BGL) enzyme molecules in UiO-66-NH_2_ and ZIF-8 via a ball milling method, modified based on our previous report^32^. (For the detailed grinding procedures, see the Methods section.) BGL catalyzes the hydrolysis of cellobiose into glucoses, which is a critical step in biomass production^41,42^. We introduced both MOF precursors and lyophilized BGL enzyme into a zirconia grinding jar and ground at a frequency of 8 Hz for 5 min in a Retsch MM400 Mixer Mill machine. The resulting powder was collected and washed by transferring it to a 50 mL vial containing 0 °C deionized water and stirring for 1 h. The synthesized samples are hereafter denoted as BGL@UiO-66-NH_2_ and BGL@ZIF-8. Both were first examined by powder x-ray diffraction (PXRD) to identify crystal structures. The appearance of the characteristic peaks of ZIF-8 and UiO-66-NH_2_ (Fig. 2a and Supplementary Fig. 1) indicates that the introduction of enzymes during the ball milling procedure does not inhibit the formation of MOF crystals. To analyze whether the enzymes were encapsulated in the frameworks, we digested the samples and analyzed them with sodium dodecyl sulfate–polyacrylamide gel electrophoresis (SDS–PAGE). Both BGL@UiO-66-NH_2_ and BGL@ZIF-8 clearly show a band indicative of BGL integration. Focusing first on the BGL@UiO-66-NH_2_, as shown in Fig. 2b, the band corresponding to the molecular weight of monomeric BGL, 65 kDa, is clearly seen for digested BGL@UiO-66-NH_2_, similar to free BGL, indicating that the enzymes were encapsulated during the ball milling process. To exclude the possibility that BGL molecules were adsorbed on the external surface of the MOF crystals, a control experiment was prepared by physically mixing the BGL with pure UiO-66-NH_2_ (synthesized by the mechanochemical method). In brief, 25 mg UiO-66-NH_2_ samples were introduced into a 10 mL vial containing 0 °C tris buffer (50 mM, pH 7.0) with BGL (1.0 mg mL^−1^), stirred for 30 min, and vacuum dried at room temperature. After the same processes of cleaning and digestion (For the detailed washing procedures, see the Methods section), no bands were observed for BGL-on-UiO-66-NH_2_, which indicates that the interaction between the MOF external surface and the enzyme molecules is not strong. The loading of BGL is controlled by the amount of enzyme introduced during the ball milling process and was characterized by a standard Bradford assay method. The loading was controlled to be ∼13.5 wt% for the following catalysis study. The same method has indicated successful BGL@ZIF-8 formation (Supplementary Fig. 2). To reveal the distribution of enzymes in UiO-66-NH_2_, we performed confocal microscopy on a sample of enzyme@UiO-66-NH_2,_ in which the BGL is labeled with fluorescent tag^43^ (FITC-BGL@UiO-66-NH_2_) (Supplementary Figs. 3 and 4). The fluorescence images show that the enzyme molecules are distributed evenly throughout the MOF crystal. We have also prepared a control sample by physically mixing FITC-labeled beta-glucosidase (FITC-BGL) with UiO-66-NH_2_ particles (FITC-BGL-on-UiO-66-NH_2_). It can be seen that, in these samples, enzymes are only distributed on the surface.

### Studying activity

**Fig. 3:**
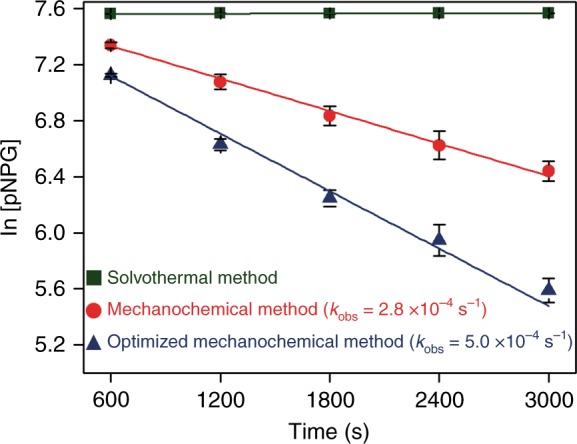
The biological activity of BGL@UiO-66-NH_2_. The biocomposites synthesized via the optimized two-step mechanochemical method (blue), one-step mechanochemical method (red), and solvothermal method (olive). Error bars are standard deviations (*n* = 3). Source data are provided as a Source Data file

With indication of enzyme encapsulation, the question remained whether the encapsulated enzymes retained their desired functionality. We thus carried out the hydrolysis of one of cellobiose’s analogs, 4-nitrophenyl β-d-glucopyranoside (pNPG), to 4-nitrophenol (pNP) over our samples. The absorbance at 405 nm of pNPG is monitored as a measure of reaction progress (see Supplementary Fig. 5). Chosen amounts of sample were dispersed into 0.5 mL of buffer (pH 6.0, 20 mM). After incubation at 37 °C for 30 min, the activity was assessed by adding 0.5 mL of 4 mM pNPG, letting the reaction run for the desired amount of time, and then terminating the reaction by pipetting 50 μL of the solution into 950 μL of a NaOH–glycine buffer (0.4 M, pH 10.8). As shown in Fig. 3, apparent biological activity was observed for the sample synthesized via the mechanochemical method (*k*_obs_ = 2.8 × 10^–4^ s^−1^), indicating that the mechanochemical process allows enzymatic functionality to be preserved (Supplementary Figs. 6 and 7). As a comparison, we also used the conventional solution-based de novo method to encapsulate BGL into UiO-66-NH_2_. (It should be noted that, to our knowledge, there are no previous reports of any solution-based method for synthesizing an enzyme@UiO-66 composite; rather, we developed the solution-based encapsulation method based on both our previous de novo strategy^16^ and a reported method for obtaining UiO-66^44^). As expected, the composite catalysts synthesized via this solution-based method showed no biological activity. We characterized the sample via the same procedures used on the mechanochemically encapsulated sample. The characteristic PXRD peaks of UiO-66-NH_2_ were observed and the enzyme loading was calculated to be ~15.1 wt% through the standard Bradford assay method. However, no band was observed for the solution-based samples (Supplementary Figs. 8 and 9). This observation fits our hypothesis that the organic solvent and high temperature denature and degrade the protein^45,46^. That is to say, we observed reasonable loading via the Bradford assay, but the SDS–PAGE band was not observed. (Note that as long as enzyme fragments are encapsulated, a loading percentage can be calculated without a band on the SDS–PAGE gel. The Bradford assay we used is conducted by binding dye onto the specific amino group and changing the dye absorbance, from which loading is calculated. Degradation of the enzyme will not necessarily affect these amino groups.) This observation demonstrates that the enzyme molecules were encapsulated into UiO-66-NH_2_ via the solution-based method but, as expected, the integrity was destroyed and the biofunctionality was lost.

### Tuning synthetic conditions

After we demonstrated that the mechanochemical method is suitable for the encapsulation of enzymes, we studied the effect of changing the enzyme addition point during the ball milling process. We hypothesized that, although we only introduced a small amount during the mechanochemical encapsulation, solvent could still impact the enzymatic activity. It is then possible that the biological activity of BGL could be further increased if contact between the enzyme and solvent could be further reduced. To test our hypothesis, we divided our mechanochemical process into two steps. The necessary nominal amount of ethanol is introduced during MOF seed formation, so we first initiated the ball-milling process to form UiO-66-NH_2_ seeds without enzyme. Then, the enzymes and more MOF precursors were added without addition of more solvent. Because ethanol is volatile, it evaporates during the first seeding step. We investigated the effect of adding the enzyme at different time points (Supplementary Fig. 10). We introduced the enzyme molecules at 1 min, 2.5 min, and 4 min after the initiation of the ball-milling process and compared the samples. We found that the 2.5-min case provides a good balance between activity and structure (Figs. 2a and 3 and Supplementary Figs. 10–13)^47,48^. An overall synthesis time of 5 min was chosen for ease of industrial application and fixed hereafter. The amount of BGL loading on BGL@UiO-66-NH_2_ was controlled to be ∼15.5 wt%, characterized by a standard Bradford assay method, which is similar to the original one-step mechanochemical method result of ∼13.5 wt%. As compared to the original one-step method, the two-step method yields a more active composite catalyst with a *k*_obs_ of 5.0 × 10^−4^ s^−1^ (Fig. 3 and Supplementary Fig. 14 and Supplementary Table 1). This result supports our hypothesis that the two-step mechanochemical encapsulation process is able to improve biological activity.

### Comparing enzyme encapsulation in different MOFs

**Fig. 4:**
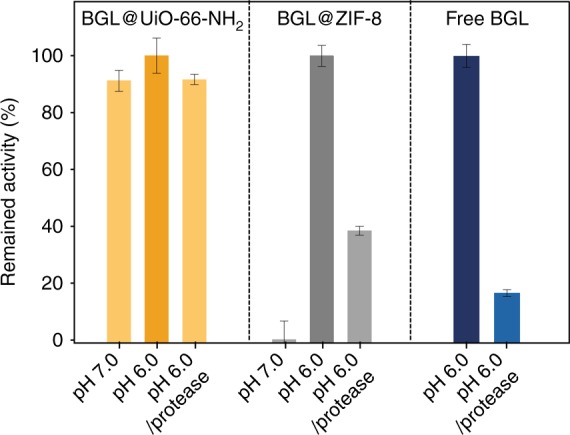
Biological activity of BGL@MOFs and free BGL. The BGL@UiO-66-NH_2_, BGL@ZIF-8, and free BGL at neutral conditions, acidic conditions, and acidic conditions with protease treatment. For the protease treatment, BGL@MOF samples were incubated with protease under acidic conditions for 2 h at 37 °C and then activity was characterized by assaying at the same temperature. The decomposition of ZIF-8 composites was observed at pH levels below 6.0. Error bars are standard deviations (*n* = 3), except for BGL@ZIF-8 at pH = 6.0 (*n* = 2). Source data are provided as a Source Data file

**Fig. 5:**
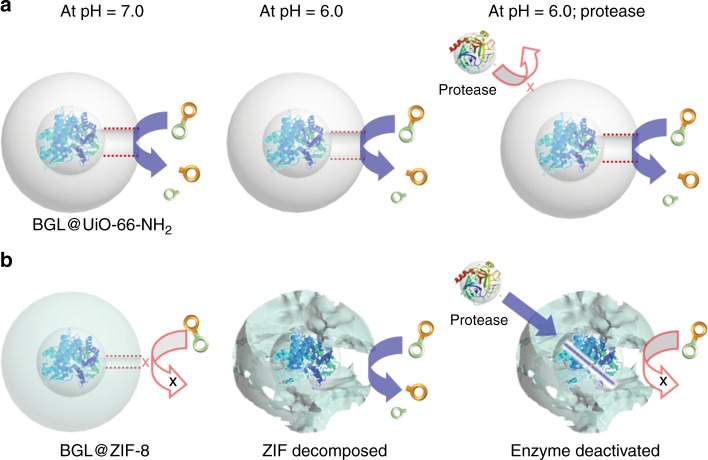
Schematic illustrations of the composite structure change. **a** BGL@UiO-66-NH_2_ and **b** BGL@ZIF-8 biocomposites are under different conditions

It is important to highlight that our mechanochemical method can be extended to a variety of MOFs, allowing it to satisfy broader demand. As mentioned above, only ZIF-type MOFs were demonstrated to encapsulate enzymes via the solution-based de novo method^15,23,49,50,51^. However, in many applications, ZIF-type MOFs, such as ZIF-8 and ZIF-90, have limited efficacy. For example, BGL normally catalyzes reactions under acidic conditions, which can cause ZIF-8 and ZIF-90 to break down, and BGL needs a larger aperture size due the substrate size of the pNPG (ZIF-8 aperture: 3.5 Å; pNPG dimensions: 5.4 Å × 6.0 Å). UiO-66, on the other hand, has an aperture size of ~6.0 Å and is stable in acidic conditions. As proof-of- concept, BGL@UiO-66-NH_2_ and BGL@ZIF-8 were both subjected to two catalytic reaction conditions and compared (Figs. 4 and 5; Supplementary Figs. 12 and 15). For the first comparison, the aperture size limitation is tested under a neutral environment. PXRD results after reaction indicate that the MOF structures of both BGL@UiO-66-NH_2_ and BGL@ZIF-8 are maintained (Supplementary Fig. 16). As expected, BGL@ZIF-8 did not display any obvious activity, likely because the pore size of ZIF-8 is not large enough for the substrate to reach BGL through the framework. Activity is, however, observed for BGL@UiO-66-NH_2_ (*k*_obs_ = 5.0 × 10^−4^ s^−1^). To verify that the lack of activity in BGL@ZIF-8 is a result of the ZIF-8 pore size, and not structural damage to BGL during ZIF-8 encapsulation, the encapsulated BGL was tested by digesting the ZIF-8 (pH ~ 6.0) and measuring the activity of BGL after ZIF-8 removal. As shown in Supplementary Fig. 17, BGL showed an observed rate constant (*k*_obs_) of 1.9 × 10^−4^ s^−1^, demonstrating that the enzyme molecules survived the grinding process. For the second comparison, the environmental limitation is tested under an acidic condition (pH 6.0) and in the presence of protease, which can hydrolyze peptide bonds and deactivate BGL. The protease we used is a mixture of at least three proteolytic enzymes, with size ranging from 16 kDa to 27 kDa, to ensure hydrolysis efficiency. Three samples, free BGL, BGL@UiO-66-NH_2_, and BGL@ZIF-8, were tested. As shown in Fig. 4, free BGL retained only 16% of its activity after protease exposure. In contrast, the activity of BGL@UiO-66-NH_2_ showed no significant decay after a 2 h incubation with protease (pH 6.0) because UiO-66-NH_2_ is stable under acidic conditions and protects BGL from protease, which is much larger than the MOF aperture size (6.0 Å). BGL@ZIF-8 shows lower biological activity under this condition since ZIF-8 is unstable at this pH level. When ZIF-8 breaks down, BGL is exposed and hydrolyzed by protease, destroying its activity (Fig. 5).

It is worth noting that the majority of BGL molecules are indeed encapsulated within ZIF-8 after the synthesis. The trace amount of surface adsorbed BGL molecules of BGL@ZIF-8 and BGL-on-ZIF-8 samples were removed after our washing procedure. Besides MOF aperture size selectivity, the activities of the enzyme molecules could also be decreased owing to the hydrophobic surface of ZIF-8^23^. We believe it further demonstrates that it is important to have the capability to encapsulate enzymes into different MOFs because different MOFs could have different effects on the enzymes. Our ball milling approach expands the selection of MOFs, which allows us to use the proper MOFs for different enzymes and catalytic reactions.

### Extending encapsulation scope

After establishing the ability of UiO-66-NH_2_ and ZIF-8 to encapsulate BGL, the scope of the mechanochemical method was expanded by testing different enzyme-MOF combinations. In total, 270 kDa invertase (Inv)^52^ and 105 kDa β-galactosidase (β-gal) were chosen to be encapsulated into UiO-66-NH_2_ because their large difference in molecular weight highlights the generality of the developed method. Both enzymes are widely used for food processing as artificial sweeteners^53–58^. After encapsulation, the resulting Inv@UiO-66-NH_2_ and β-gal@UiO-66-NH_2_ samples were studied by PXRD analysis and Bradford assays (Supplementary Figs. 18 and 19). The enzyme loading in Inv@UiO-66-NH_2_ and β-gal@UiO-66-NH_2_ were ∼14.8 and 12.3 wt%, respectively, similar to that of BGL@UiO-66-NH_2_. In addition, the embedded enzymes showed good biological activity (Supplementary Fig. 20). As shown in Supplementary Figs. 21 and 22, the encapsulated Inv molecules maintained their biological activity under the aforementioned protease conditions, whereas the activity of free Inv decreased to ~1% of its original value under the same conditions. This suggests that the embedded large Inv molecules were effectively shielded against proteolysis by the UiO-66-NH_2_.

Catalase (CAT) was chosen to be encapsulated into ZIF-8 as it catalyzes hydrogen peroxide dissociation and hydrogen peroxide is smaller than the ZIF-8 aperture size, allowing the substrate to reach CAT. After encapsulation, the formation of ZIF-8 and enzyme loading were examined by PXRD analysis and Bradford assay (Supplementary Fig. 23). The surface-adsorbed CAT was removed via a washing procedure described in the Methods section^59^. To demonstrate that ZIF-8 could protect the enzymes, CAT@ZIF-8 was incubated with proteinase K for one hour and the biological activity assays showed an observed rate constant (*k*_obs_) of 2.5 × 10^–4^ s^−1^ (Supplementary Fig. 24). This corroborates the previous results with BGL, demonstrating that enzymes encapsulated in ZIF-8 retain their biological activity as well as possess size-sheltering functionality.

To demonstrate the generality of the method, CAT was encapsulated in Zn-MOF-74 via the ball milling process. Zn-MOF-74, a member of the M-MOF-74 (CPO-27) family, is formed with stoichiometric ZnO and 2,5-dihydroxyterephthalic acid (H4dhta)^60^. The synthesized samples were examined by PXRD to identify crystal structures (Supplementary Fig. 25). Prior to the biological activity assays, all as-synthesized enzyme@MOF composites were incubated in pH 8.0 Tris buffer with proteinase K to remove residual CAT on the MOF surface. CAT@Zn-MOF-74, with an enzyme loading of ~8%, showed a notably high observed rate constant of 3.55 × 10^–2^ s^−1^ (Supplementary Fig. 26). These factors reduced the impact of the ball milling process on enzymatic activity. Overall, the developed method can be generally applied to encapsulate enzymes of various sizes into MOFs with varied structures.

## Discussion

We demonstrated the encapsulation of enzymes into MOFs via a ball milling process. We applied this technique to BGL, encapsulating it in both UiO-66 and ZIF-8 and showing that it retained its catalytic activity. We tuned our synthetic process by introducing enzymes at different time points during the synthesis to increase post-synthetic enzymatic activity. We investigated the effect of pH and protease on our composite systems, reinforcing that robust MOFs like UiO-66-NH_2_ can protect enzymes like BGL from unfavorable biological conditions. The scope of the developed technique was expanded to include a variety of enzymes, with varied sizes and catalytic reactions, and MOFs, with varied structures. To our knowledge, this method represents the report of the mechanochemical encapsulation of biocatalysts into MOFs, including the overall encapsulation method for enzymes into UiO-66-type MOFs, and the insights presented herein can be extended to generate robust and recyclable biocomposites for use in a number of industrial applications.

## Methods

### Synthesis of zirconium(IV) oxo hydroxy methacrylate cluster

The cluster was synthesized according to a previously reported method^39^ with minor modifications. Methacrylic acid (1.4 mL) was added to 2 mL Zr(OPr)_4_ solution (70% solution in n-propanol), followed by the addition of a drop of water. The reaction mixture was heated in an oven at 60 °C for 3 days. The formed colorless solid was filtered under vacuum and washed once with 2 mL of 2-propanol. The product was subjected to PXRD prior to further work (Supplementary Fig. 27).

### Synthesis of UiO-66-NH_2_ by LAG

The formation of UiO-66-NH_2_ through liquid-assisted grinding (LAG) was carried out in optimized conditions in 25 mL zirconia jars with a 3.5 g zirconia ball on a Retsch MM400 Mixer Mill operated with a mechanofrequency of 8 Hz. Typically, zirconium(IV) oxo hydroxy methacrylate (25 mg, 0.0147 mmol) and 2-aminoterephthalic acid (16 mg, 0.0882 mmol) were prepared and added into the grinding jar in two steps. First, half the desired precursor amount (including zirconium(IV) oxo hydroxy methacrylate and 2-aminoterephthalic acid) was placed into the jar. Then, 41 μL of ethanol was added as the assisting liquid. After 2.5 min (150 s) of grinding at 8 Hz to form the crystal seeds, the rest of the precursors were introduced into the jar and the synthesis was completed with an additional 2.5 min (150 s) of grinding at the same frequency. The as-synthesized samples were centrifuged, washed with deionized water three times, and vacuum dried at room temperature.

### One-pot synthesis of BGL@UiO-66-NH_2_

One-step BGL@UiO-66-NH_2_ was prepared through a method similar to that of UiO-66-NH_2_. Zirconium(IV) oxo hydroxy methacrylate (25 mg, 0.0147 mmol), 2-aminoterephthalic acid (16 mg, 0.0882 mmol), and BGL (10 mg) were prepared and added into a grinding jar, followed by the addition of 41 μL of ethanol as the assisting liquid. After 5 min of grinding at 8 Hz, the as-synthesized samples were centrifuged, washed with deionized water three times, stirred with 15 mL of deionized water for 1 h, and vacuum dried at room temperature. All samples were synthesized at room temperature and stored at 4 °C for further use.

### One-pot synthesis of CAT@Zn-MOF-74

Zinc oxide (36 mg, 0.44 mmol) was mixed with CAT (20 mg) and 1 mL of deionized water in an Eppendorf tube. Thereafter, 2,5-dihydroxyterephthalic acid (44 mg, 0.22 mmol) and 50 μL DMSO (25 vol%) were placed into the milling jar and ground at a mechanofrequency of 15 Hz for 15 min. The as-synthesized samples were subsequently centrifuged and quickly washed with 5 mL of deionized water three times. To remove enzyme residues on the MOF surfaces, these samples were again washed in a 10 mL vial containing 0 °C tris buffer (50 mM, pH 8.0) with proteinase K (0.1 mg mL^−1^), stirred for 30 min, and vacuum dried at 25 °C room temperature. The sample was then stored at 4 °C for further use. The protein loading of CAT@Zn-MOF-74 was determined to be ∼8.6 wt% by the standard Bradford assay method.

### Two-step synthesis of Glycosidases@UiO-66-NH_2_

The two-step synthesis method is again similar to that of UiO-66-NH_2_. Zirconium(IV) oxo hydroxy methacrylate (25 mg, 0.0147 mmol) and 2-aminoterephthalic acid (16 mg, 0.0882 mmol) were prepared and added into a grinding jar in two steps. First, half the desired precursor amount was placed into the jar. In all, 41 μL of ethanol was then added as the assisting liquid. After 2.5 min of grinding at 8 Hz, the rest of the precursors, mixed with 10 mg of the chosen glycosidase, such as BGL or Inv, were introduced into the jar and the synthesis was completed with an additional 2.5 min of grinding at the same frequency. The as-synthesized samples were centrifuged, washed with 30 mL deionized water in 50 mL centrifuge tube three times, stirred with 0 °C 25 mL of deionized water for 1 h in a 50 mL vial, and vacuum dried at room temperature (Supplementary Figs. 28 and 29). All samples were synthesized at room temperature and stored at 4 °C for further use.

### Synthesis of BGL@ZIF-8/CAT@ZIF-8 by LAG

Zinc oxide (40.7 mg, 0.5 mmol) and 2-methylimidazole (82.6 mg, 1.0 mmol) were prepared and divided into two equal portions. One portion was placed in a milling jar, 60 μL of ethanol was added as an assisting liquid, and the mixture was ground for 2.5 min at a mechanofrequency of 8 Hz. Subsequently, 10 mg of BGL/CAT was added, followed by the other precursor portion, and the mixture was ground for another 2.5 min at the same frequency. The as-synthesized BGL@ZIF-8 samples were centrifuged, washed with deionized water three times, filtered under vacuum, and washed with 60 mL of 50% EtOH_(aq)_. The sample was then vacuum dried at room temperature and stored at 4 °C for further use. The protein loading of BGL@ZIF-8 was determined to be ∼9.5 wt% using the standard Bradford assay method. The as-synthesized CAT@ZIF-8 samples were centrifuged, washed with deionized water three times, stirred with 10 mL of proteinase K solution (0.05 mg mL^−1^) for 30 min, and vacuum dried at room temperature. The sample was subsequently stored at 4 °C for further use. The protein loading of CAT@ZIF-8 was determined to be ∼2.2 wt% using the standard Bradford assay method.

### Solvothermal synthesis of BGL@UiO-66-NH_2_

The procedure was based on a previous report^44^ with slight modifications. ZrCl_4_ (125 mg) was dissolved in 5 mL of dimethylformamide (DMF) solution with 1 mL concentrated HCl solution. Then, 10 mg BGL and 134 mg 2-aminoterephthalic acid in DMF solution (10 mL) were added, and the mixture was heated in an oven at 80 °C for 10 h. The as-synthesized samples were centrifuged and washed with 15 mL of DMF three times and with 15 mL of MeOH twice. They were then centrifuged, vacuum dried at room temperature, and stored at 4 °C for further use.

### Activity of BGL

The catalytic activity of BGL was determined based on the change in concentration of pNP, the product of pNPG hydrolysis, based on a prior report^55^. The concentrations of enzyme and BGL in each trial (i.e., BGL@UiO-66-NH_2_, BGL@ZIF-8, and free BGL) were kept the same (0.3 mg BGL mL^−1^). For BGL@UiO-66-NH_2_, 3.0 mg of the composite (∼13.5 ± 0.1 wt% of BGL) were dispersed into 0.5 mL of citric buffer (pH 6.0, 20 mM) and the sample was incubated at 37 °C for 30 min. The biological activity of BGL was assayed by adding 0.5 mL of 4 mM pNPG (pH 6.0 citric buffer solution) as a substrate, and then the reaction was terminated by pipetting 50 μL of the solution into 950 μL of NaOH–glycine buffer (0.4 M, pH 10.8). The final concentration of pNP was calculated by measuring the absorbance at a wavelength of 405 nm using a Jasco V-730 ultraviolet-visible spectrophotometer. For the protease treatment, 3.0 mg of BGL@UiO-66-NH_2_ was incubated with 4 mg protease in 500 μL of a 1 mM citric buffer (pH 6.0) for 2 h prior to biological activity assays. The concentrations on the calibration curve ranged from 0 to 2 mM (Supplementary Fig. 5).

### Activity of Inv

The catalytic activity of Inv was determined from the concentration of glucose and fructose based on a PAHBAH assay^61^. The concentration of Inv was kept the same (0.3 mg Inv mL^−1^). For Inv@UiO-66-NH_2_, 2.0 mg of the composite (∼14.8 ± 1.2 wt% of Inv) were dispersed into 0.5 mL of a citric buffer (pH 4.4, 20 mM) and the sample was incubated at 37 °C for 30 min. The biological activity of Inv was assayed through the addition of 0.5 mL of 4 mM sucrose (citric buffer solution) as substrate. After the desired time, the reaction was terminated by pipetting 50 μL of the reaction solution into 950 μL of PAHBAH reagent (5 mg mL^−1^ PAHBAH in 0.5 M NaOH), heated 6 min at 95 °C, cooled 1 min at 4 °C, reheated 1 min at room temperature, and the absorbance at 410 nm was read. The concentration of the calibration curve ranged from 0 to 4 mM (Supplementary Fig. 30).

### Activity of β-gal

The catalytic activity of β-gal was determined from the concentration of 2-nitrophenol (oNP) based on a previous report^53^. The concentration of β-gal was maintained (0.075 mg β-gal mL^−1^). For β-gal@UiO-66-NH_2_, ~0.6 mg of material (∼12.5 wt% of β-gal) was dispersed in 0.5 mL citric buffer (pH 5.0, 20 mM) and incubated at 37 °C for 30 min. The biological activity of β-gal was assayed by addition of 0.5 mL of 5 mM 2-nitrophenyl β-d-galactopyranoside (oNPG) (citric buffer solution) as a substrate and the reaction was subsequently terminated by pipetting 50 μL of solution into 950 μL of Na_2_CO_3_ (1.0 M). The final concentration of oNP was calculated by measuring the absorbance at 417 nm using a Jasco V-730 ultraviolet-visible spectrophotometer. The calibration curve concentration ranged from 0 to 8 mM (Supplementary Fig. 31).

### SDS–PAGE analysis of BGL@UiO-66-NH_2_/BGL-on-UiO-66-NH_2_

Prior to SDS–PAGE analysis, both of the samples were cleaned with 30 mL of deionized water in a 50 mL centrifuge tube three times and then transferred to a 50 mL vial with 25 mL of 0 °C deionized water, stirred for 1 h, and vacuum dried. 25 mg of BGL@UiO-66-NH_2_ (or BGL-on-UiO-66-NH_2_) were dissolved in 1 mL of 3% hydrofluoric acid and 1 mL of an EDTA buffer (pH 10.0, 1.0 M). After 1 h of incubation, 10 μL of the solution were mixed with Laemmli sample buffer 2 × (125 mM Tris-HCl (pH 6.8), 4% SDS, 0.004% bromophenol blue, 20% glycerol and 10% β-mercaptoethanol), and the mixtures were electrophoresed on SDS–PAGE (4% polyacrylamide stacking gel, re-solving gel 12% acrylamide) at 80 V under reducing conditions (boiled at 95 °C in dry bath incubator for 5 min). Then, the gel was used for Commassie Blue Fast Staining according to the instruction manual.

### MOF decomposition method for enzyme@MOFs

First, 3 mg of the glycosidase@UiO-66-NH_2_ composite were dissolved in 250 μL of 0.3 M NaOH and sonicated for 30 min. The solution was subsequently mixed with 250 μL of 0.5 M HCl to obtain an appropriate pH for the Bradford assay. Enzyme@ZIF-8: 3 mg of BGL@ZIF-8 or 4 mg of CAT@ZIF-8 were dissolved in 500 μL of 0.3 M HCl and assayed. CAT@Zn-MOF-74: 3.5 mg of CAT@Zn-MOF-74 was decomposed using 400 µL of NaOH (1.875 M), neutralized with 500 µL of HCl (1 M) and subsequently assayed.

### Protease treatment of BGL@ZIF-8

Prior to the proteolytic assays, the materials were digested. It should be noted that the solution pH generally increased by ~0.5 pH units after digestion due to release of ZIF-8 linkers. First, 3.2 mg of BGL@ZIF-8 was incubated in 200 μL of 200 mM citric acid buffer (pH 5.5) for 1 h and added to 300 μL of 200 mM citric acid buffer (pH 5.5) containing 1 mg of protease for 2 h. The solution was assayed by addition of 500 μL of 4 mM pNPG in pH 6 citric acid buffer solution.

### Protease treatment of Inv and Inv@UiO-66-NH_2_

First, 0.3 mg of Inv or 2.0 mg Inv@UiO-66-NH_2_ (14.8 wt%) was incubated in 200 μL of 1 mM citric acid buffer (pH 6.0) for 1 h and added to 4 mg of protease in 300 μL of 1 mM citric acid buffer (pH 6.0) for 2 h. The solution was assayed by addition of 500 μL of 4 mM sucrose (5 mM, pH 4.4 citric buffer solution).

### Proteinase K treatment of CAT@Zn-MOF-74

First, 3.5 mg of washed CAT@MOF-74 (∼8.6 wt% CAT) was incubated in 400 μL of 50 mM Tris buffer (pH 8.0) for 30 min and added to 100 μL of 50 mM Tris buffer (pH 8.0) containing proteinase K (1.0 mg mL^−1^) for 30 min. The solution was assayed by addition of 500 μL of 200 μM H_2_O_2_ in pH 8 Tris buffer solution.

### Proteinase K treatment of CAT@ZIF-8

First, 13.6 mg of CAT@ZIF-8 (∼2.2 wt% CAT in CAT@ZIF-8) was incubated in 400 μL of 50 mM Tris buffer (pH 8.0) for 30 min and subsequently added to 100 μL of 50 mM Tris buffer (pH 8.0) with 0.05 mg proteinase K. Proteinase K incubation of CAT@ZIF-8 was performed for 1 h owing to sample treatment by proteinase K during the washing step (0.5 h). The solution was assayed by addition of 500 μL of 200 μM H_2_O_2_ in pH 8 Tris buffer solution.

### Procedures for FITC-BGL

The procedure was based on a previous report^43^ with minor modifications. An enzyme stock solution was prepared by dissolving 50.0 mg in 2.5 mL of 0.85% physiological saline solution. A fluorescein-5-isothiocyanate (FITC) solution was prepared in 0.5 M carbonate–bicarbonate buffer of pH 9.6 with a FITC concentration of 10.0 mg mL^−1^. Then, 50 μL of FITC solution was mixed with the enzyme solution and continuously stirred for 30 min. The concentration could be adjusted by fixing the ratio of FITC/enzyme (w/w) at 0.01. The resulting solution was purified using a PD-10 column (50 kDa) and washed with 0.01 M acetate buffer of pH 5.0. The purified FITC-BGL solution was then lyophilized and stored at 4 °C until further usage.

## Supplementary information

Supplementary Information

Source Data

## Data Availability

Data supporting this study are available in the article and corresponding Supplementary Information files. The raw data underlying Figs. 2b, 3, 4 as well as Supplementary Figs. 2, 5, 6, 7, 9, 10, 12, 13, 14, 15, 17, 19, 20, 21, 22, 24, 26, 30, and 31 are provided as a Source Data file. All relevant data are available from the authors upon request.
