# Supplementary material for: Rapid mechanochemical encapsulation of biocatalysts into robust metal–organic frameworks
Source: Nat Commun. 2019 Nov 1;10:5002. doi: 10.1038/s41467-019-12966-0 (PMC6825160; doi:10.1038/s41467-019-12966-0)
Supplement: Supplementary file 1 — Supplementary Information [file 41467_2019_12966_MOESM1_ESM.pdf]

Supplementary Information

Rapid Mechanochemical Encapsulation of Biocatalysts into Robust Metal–Organic Frameworks

Wei *et al*.

TABLE OF CONTENTS

Supplementary Methods 4

Supplementary Figures 5

Supplementary Figure 1. PXRD analysis of BGL@UiO-66-NH_2........................5
Supplementary Figure 2. SDS-PAGE analysis of one-step BGL@ZIF-8. 6
Supplementary Figure 3. FIB-SEM analysis of two-step BGL@UiO-66-NH_2.....7
Supplementary Figure 4. Fluorescence microscopy imaging of FITC-BGL@UiO-66-NH_2 and FITC-BGL-on-UiO-66-NH_2. 8
Supplementary Figure 5. The calibration curve for pNP. 9
Supplementary Figure 6. The biological activity of pure UiO-66-NH_2..............10
Supplementary Figure 7. The biological activity of free β-Glucosidase (BGL)..11
Supplementary Figure 8. PXRD analysis of BGL@UiO-66-NH_2......................12
Supplementary Figure 9. SDS-PAGE analysis of solvothermal BGL@UiO-66-NH_2. 13
Supplementary Figure 10. The biological activity of BGL@UiO-66-NH_2 synthesized under different conditions. 14
Supplementary Figure 11. PXRD patterns of BGL@UiO-66-NH_2 obtained with different precursor addition times. 15
Supplementary Figure 12. SDS-PAGE analysis of two-step (2.5-2.5) BGL@UiO-66-NH_2. 16
Supplementary Figure 13. The biological activity of free β-Glucosidase (BGL) ground at 8 Hz for 2.5 min. 17
Supplementary Figure 14. The kinetic parameters of BGL in BGL@UiO-66-NH_2. 18
Supplementary Figure 15. SDS-PAGE analysis of two-step BGL@ZIF-8. 19
Supplementary Figure 16. PXRD analysis of BGL@UiO-66-NH_2 and BGL@ZIF-8 treated with neutral buffer. 20
Supplementary Figure 17. The biological activity of BGL@ZIF-8. 21
Supplementary Figure 18. PXRD analysis of glycosidases@UiO-66-NH_2. 22
Supplementary Figure 19. Bradford assay of the Inv concentration. 23
Supplementary Figure 20. The biological activity of β-gal@UiO-66-NH_2.........24
Supplementary Figure 21. The biological activity of Inv@UiO-66-NH_2............25
Supplementary Figure 22. The relative activity of samples incubated with protease. 26
Supplementary Figure 23. PXRD analysis of CAT@ZIF-8. 27
Supplementary Figure 24. The biological activity of CAT@ZIF-8. 28
Supplementary Figure 25. PXRD analysis of CAT@Zn-MOF-74. 29

Supplementary Figure 26. The biological activity of CAT@Zn-MOF-74. 30
Supplementary Figure 27. PXRD analysis of MOF-precursor zirconium clusters. 31
Supplementary Figure 28. ^{1}H NMR spectrum of BDC-NH_2. 32
Supplementary Figure 29. ^{1}H NMR spectrum of the supernatant of BGL@UiO-66-NH_2 after washing. 33
Supplementary Figure 30. Concentration of glucose. 34
Supplementary Figure 31. Concentration of oNP. 35
Supplementary Tables 36
Supplementary Table 1: The kinetic parameters on β-glucosidase in BGL@UiO-66-NH_2 36
Supplementary References 37

Supplementary Methods

All chemicals were obtained commercially and used without additional purification. Citric acid monohydrate (≥99.5%), 4-nitrophenyl β-D-glucopyranoside (pNPG, ≥98%), 2-nitrophenyl β-D-galactopyranoside (oNPG, ≥98%), β-glucosidase from almonds (BGL), invertase from baker's yeast (Inv), β-galactosidase (β-Gal) from Aspergillus oryzae, Catalase (CAT) from bovine liver, Proteinase K from Tritirachium album, Methacrylic acid (99%), $Zr(OPr)_4$ solution (70% solution in *n*-propanol), protease from Streptomyces griseus, zirconium(IV) oxo hydroxy methacrylate, acetic acid (CH_3COOH, 99%), sample buffer (Laemmli 2× concentrate) and 4-hydroxybenzoic acid hydrazide (PAHBAH, ≥97%), Dimethyl sulfoxide-d_6 (DMSO-d_6, 99.9 atom% D) were purchased from Sigma-Aldrich. 4-Nitrophenol (pNP, 99%) and 2,5-Dihydroxyterephthalic acid, (H_4dhta, 97%) were purchased from Alfa Aesar. Sodium hydroxide (NaOH, 97.0%), sodium acetate (CH_3COONa, 98%), sodium hydrogen carbonate ($NaHCO_3$, 99.5%), sodium chloride (NaCl, 99.5%) and sodium carbonate (Na_2CO_3, 99.5%) were purchased from SHOWA. Anhydrous ethanol (EtOH, >99.5%) and 2-propanol were purchased from ECHO. Sucrose was purchased from AENCORE. 2-Aminoterephthalic acid (BDC-NH_2, >98%), Fluorescein-5-Isothiocyanate (FITC, >95.0%) were purchased from Tokyo Chemical Industry. Tris(hydroxymethyl)aminomethane (Tris, >99.0%), anhydrous D-glucose (dextrose), Bradford reagent, Coomassie brilliant blue R-250, acryl/bisTM 40.0 % solution, sodium dodecyl sulfate (SDS, 20.0%), ammonium persulfate (APS, >98.0%), tetramethylethylenediamine (TEMED, >97.0%), citric acid trisodium salt dehydrate and glycine were purchased from Amresco. Hydrochloric acid (HCl, 37%) and hydrofluoric acid (HF, 48-50 w/w %) were purchased from Fisher Scientific. Zirconium (IV) chloride ($ZrCl_4$, 98%), 2-methylimidazole (mIm, 99%) and ethylenediaminetetraacetic acid (EDTA, 99%), 2-nitrophenol (oNP, 99%) were purchased from Acros Organics. Zinc oxide 20 nm nano grade was purchased from UniRegion Bio-Tech. Methanol (MeOH, 99.8%) was purchased from Scharlau. N,N-Dimethylformamide (DMF) was purchased form Tedia. Dimethyl sulfoxide (DMSO, ≥99.9%) was purchased from Merck.

Supplementary Figures

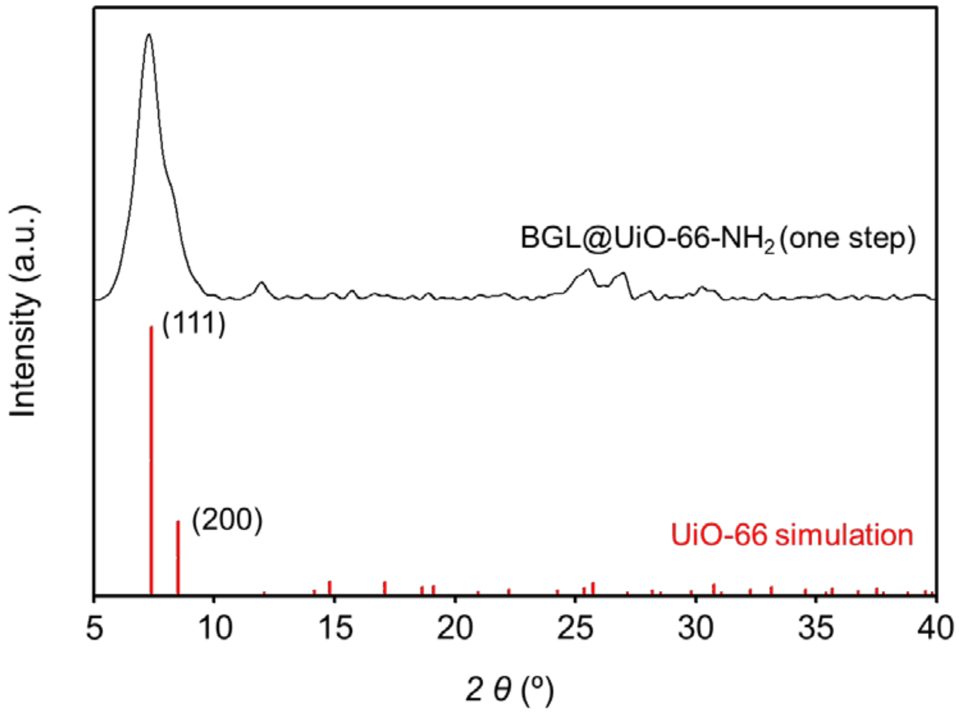

Supplementary Figure 1. PXRD analysis of BGL@UiO-66-NH$_2$.

Black line: PXRD results for BGL@UiO-66-NH$_2$ obtained in the one-step mechanochemical method showing the appearance of a (111) peak and a (200) shoulder, corresponding to UiO-66 and representing the completeness of MOF formation. Red bars: simulated peaks characteristic of UiO-66.

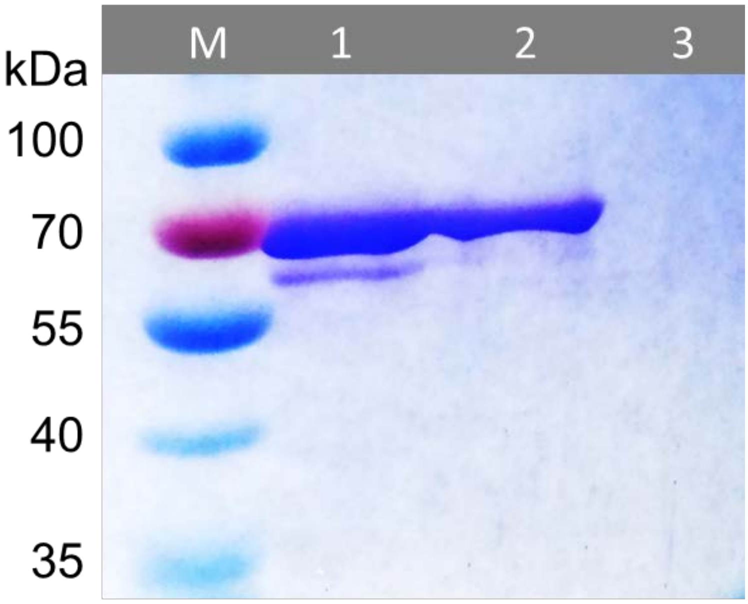

Supplementary Figure 2. SDS-PAGE analysis of one-step BGL@ZIF-8.

M: protein marker; lane 1: free BGL; lane 2: BGL@ZIF-8 (washed); lane 3: BGL-on-ZIF-8 (washed). Free BGL and BGL@ZIF-8 showed a similar band between 55 kDa and 70 kDa. No band was found in BGL-on-ZIF-8 indicating that after washing no BGL was adsorbed on the particle surface. Source data are provided as a Source Data file.

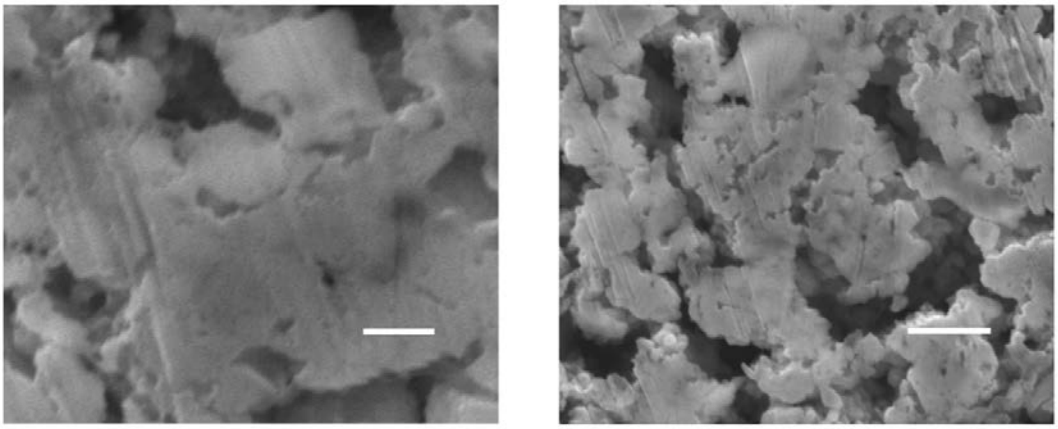

Supplementary Figure 3. FIB-SEM analysis of two-step BGL@UiO-66-NH_2.
The scale bar corresponds to left: at 500 nm magnification; right: at 1 μm magnification.

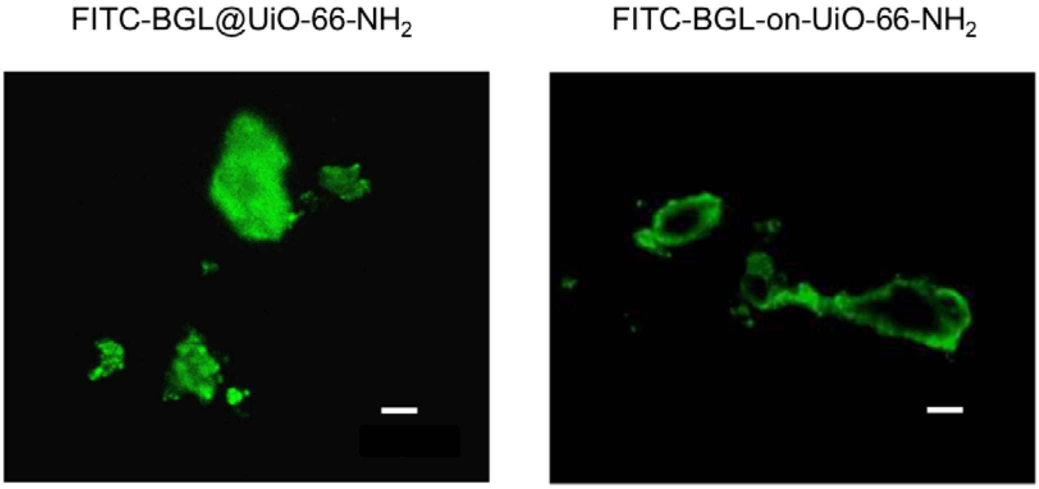

Supplementary Figure 4. Fluorescence microscopy imaging of FITC-BGL@UiO-66-NH_2 and FITC-BGL-on-UiO-66-NH_2.

The scale bars correspond to at 5 μm magnification.

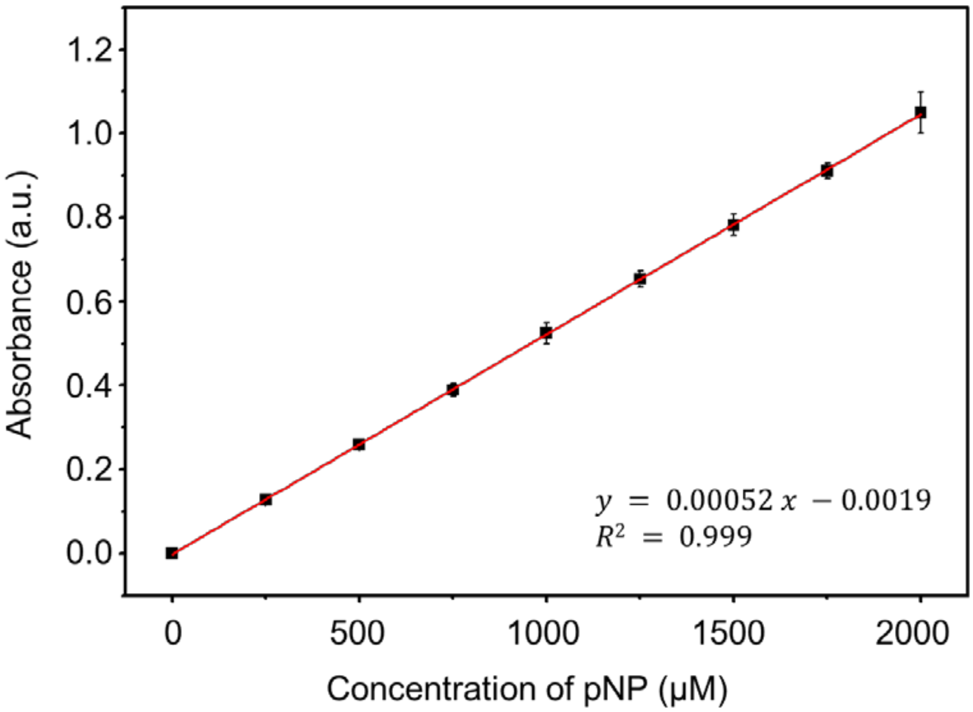

Supplementary Figure 5. The calibration curve for pNP.

The corresponding standard calibration line of the Bradford assay. Error bars are standard deviations ($n = 3$). Source data are provided as a Source Data file.

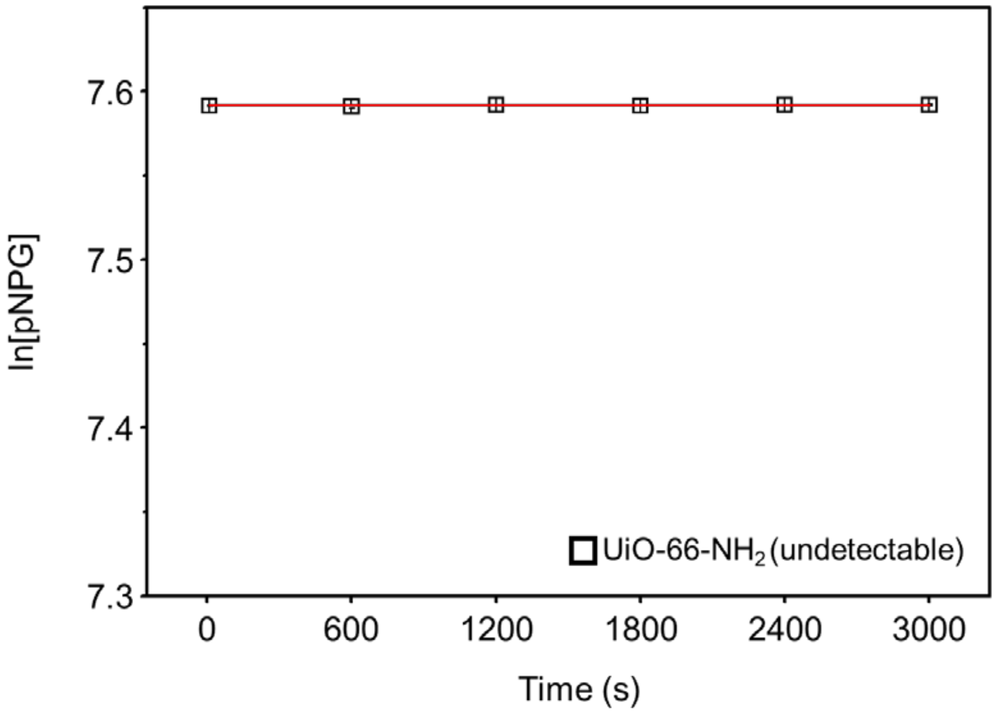

Supplementary Figure 6. The biological activity of pure UiO-66-NH_2.
The assay was performed by introducing 3.0 mg of UiO-66-NH_2 and 2 mM pNPG in 1.0 mL of citric buffer (pH 5.5, 20 mM). Error bars are standard deviations (n = 3). Source data are provided as a Source Data file.

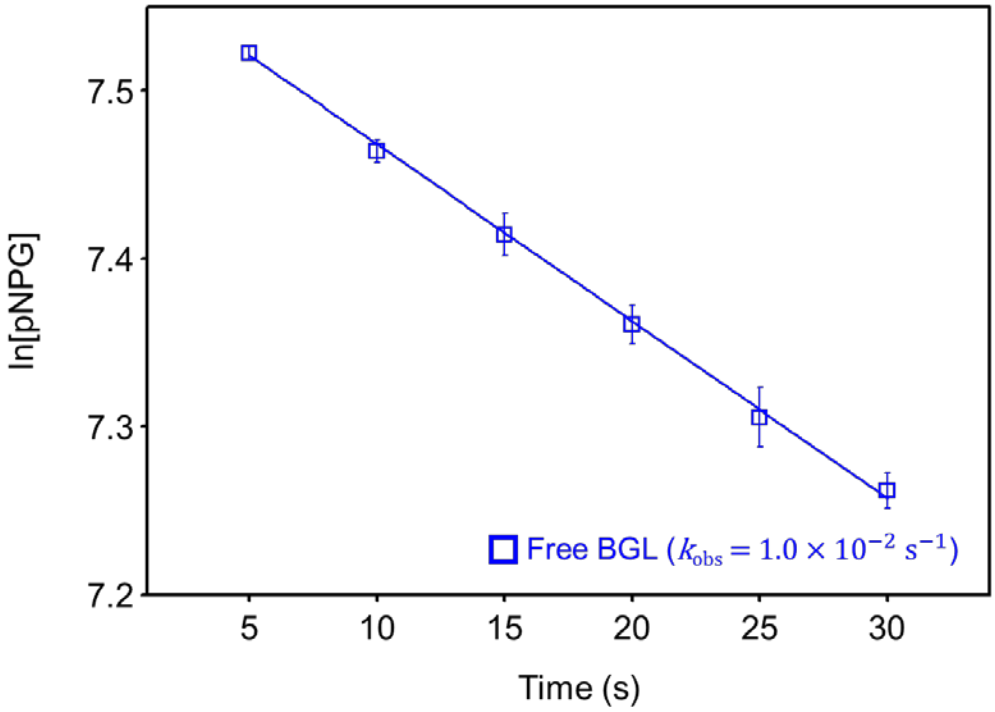

Supplementary Figure 7. The biological activity of free β-Glucosidase (BGL). The error bars indicate the standard deviation of three independent measurements. Error bars are standard deviations (n = 3). Source data are provided as a Source Data file.

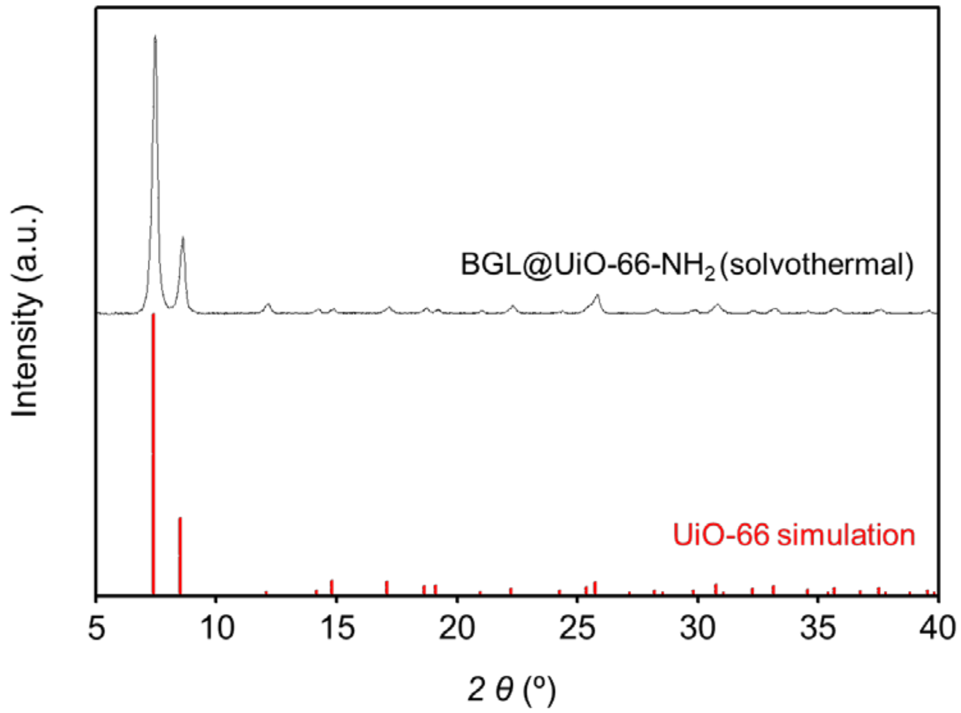

Supplementary Figure 8. PXRD analysis of BGL@UiO-66-NH_2.

Black line: PXRD results for BGL@UiO-66-NH_2, obtained through the solvothermal method, showing the completeness of MOF formation. Red bars: A simulated spectrum characteristic of UiO-66.

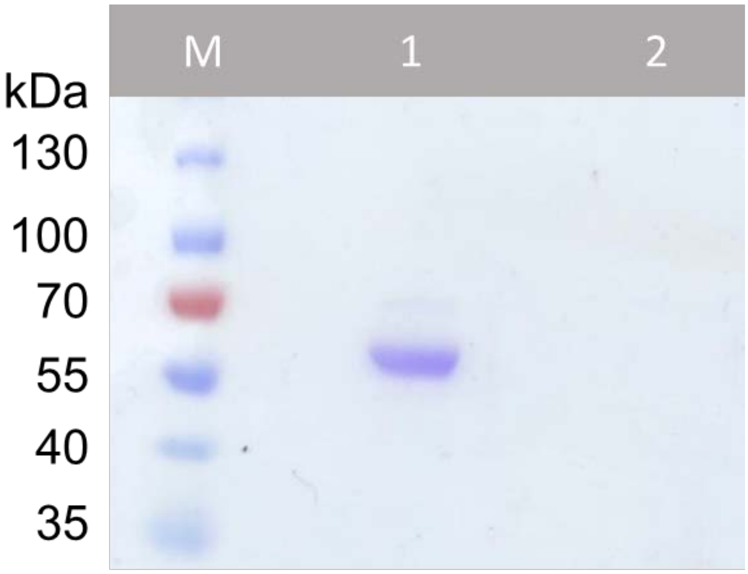

Supplementary Figure 9. SDS-PAGE analysis of solvothermal BGL@UiO-66-NH_2. M: protein marker; lane 1: free BGL; lane 2: solvothermal BGL@UiO-66-NH_2. The BGL monomer was ~65 kDa and no band was observed for the solvothermally generated BGL@UiO-66-NH_2. Source data are provided as a Source Data file.

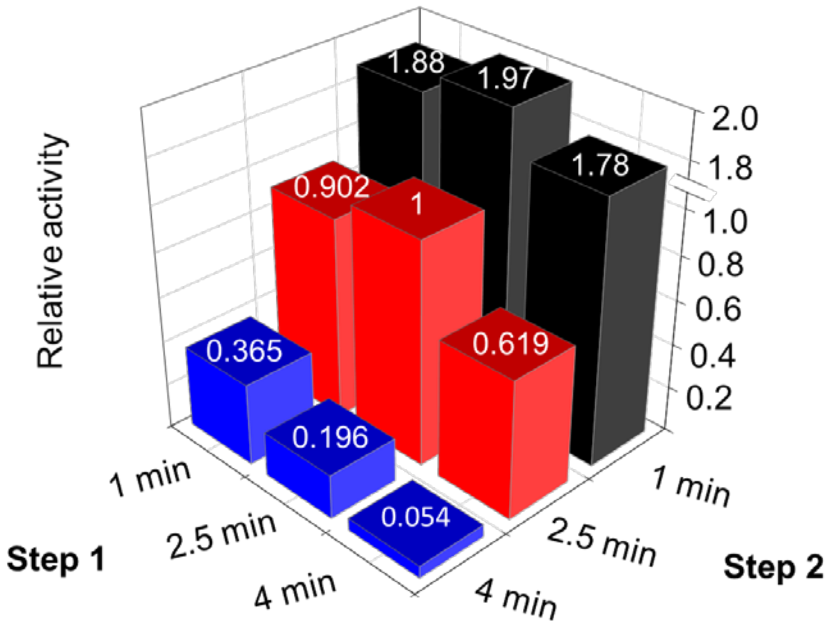

Supplementary Figure 10. The biological activity of BGL@UiO-66-NH_2 synthesized under different conditions.

Both step one and step two used three time durations, 1 min, 2.5 min, and 4 min, as a control factor to find the optimized condition. For clarity, the color of those bars represent the time duration of the second step. Source data are provided as a Source Data file.

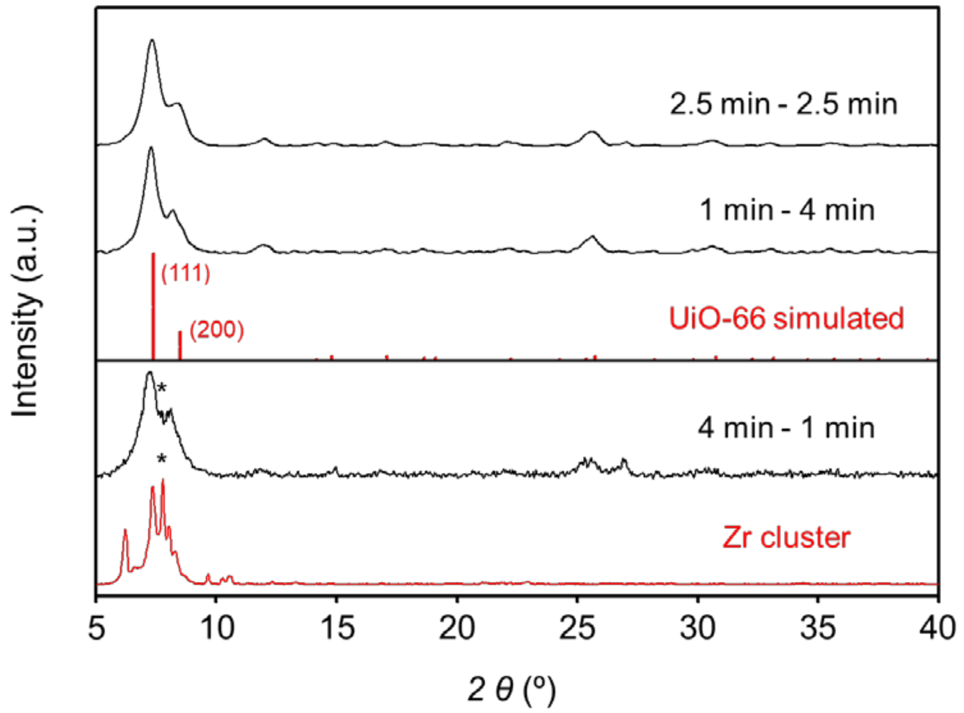

Supplementary Figure 11. PXRD patterns of BGL@UiO-66-NH$_2$ obtained with different precursor addition times.

PXRD patterns showing the difference in crystallinity seen with different precursor addition times. For the 2.5 min - 2.5 min and 1 min - 4 min line, the appearances of (111) peak and (200) peaks represent the completeness of MOF syntheses. Asterisk shown in the 4 min -1 min experiment represents the remaining Zr-cluster precursor.

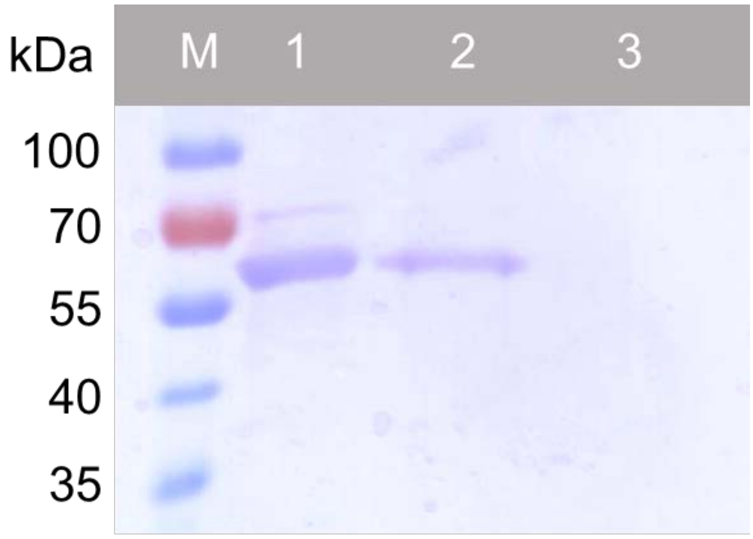

Supplementary Figure 12. SDS-PAGE analysis of two-step (2.5-2.5) BGL@UiO-66-NH_2.

M: protein marker; lane 1: free BGL; lane 2: BGL @UiO-66-NH_2 (washed); lane 3: BGL-on-UiO-66-NH_2 (washed). Free BGL and BGL@UiO-66-NH_2 showed a similar band between 55 kDa and 70 kDa. No band was found in BGL-on-UiO-66-NH_2 indicating that after washing no BGL remained adsorbed on the MOF surface. Source data are provided as a Source Data file.

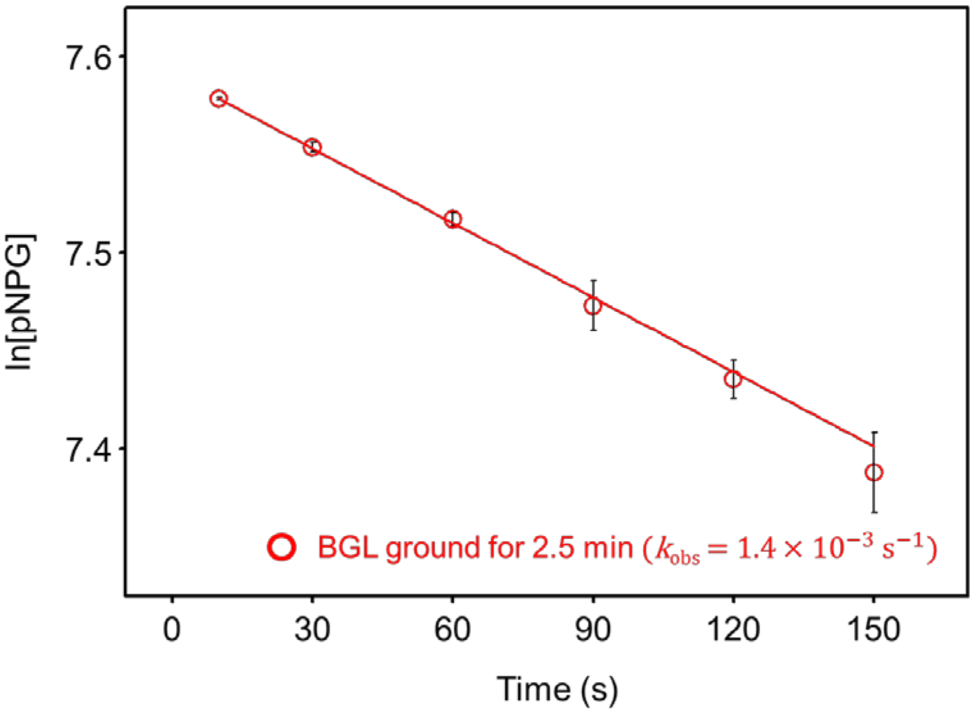

Supplementary Figure 13. The biological activity of free β-Glucosidase (BGL) ground at 8 Hz for 2.5 min.

Error bars are standard deviations (n = 3). Source data are provided as a Source Data file.

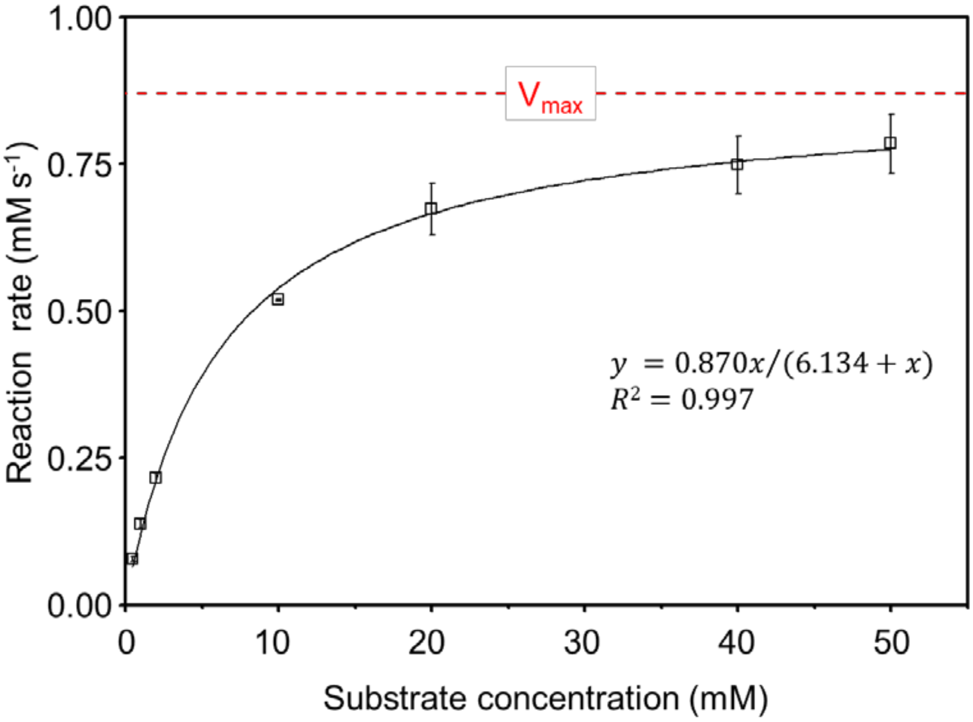

Supplementary Figure 14. The kinetic parameters of BGL in BGL@UiO-66-NH$_2$. Determination of V_{max}, K_M and k_{cat} from a plot of the reaction velocity, V, against substrate concentration [pNPG] for BGL@UiO-66-NH$_2$ in citric buffer (pH 6.0, 20 mM). Error bars are standard deviations (n = 2). Source data are provided as a Source Data file.

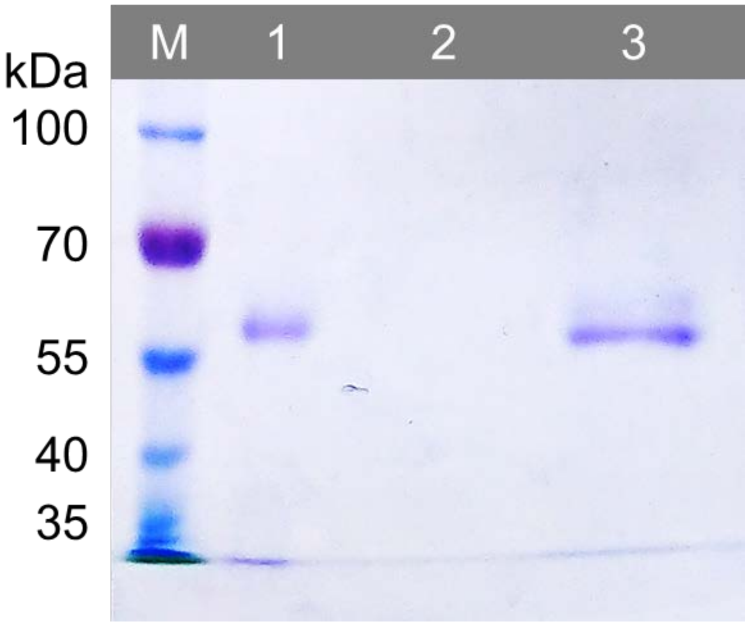

Supplementary Figure 15. SDS-PAGE analysis of two-step BGL@ZIF-8.

M: protein marker; lane 1: free BGL; lane 2: BGL-on-ZIF-8 (washed); lane 3: BGL@ZIF-8 (washed). Free BGL and BGL@ZIF-8 showed a similar band between 55 kDa and 70 kDa. No band was found in BGL-on-ZIF-8 indicating that after washing no BGL remained adsorbed on the particle surface. Source data are provided as a Source Data file.

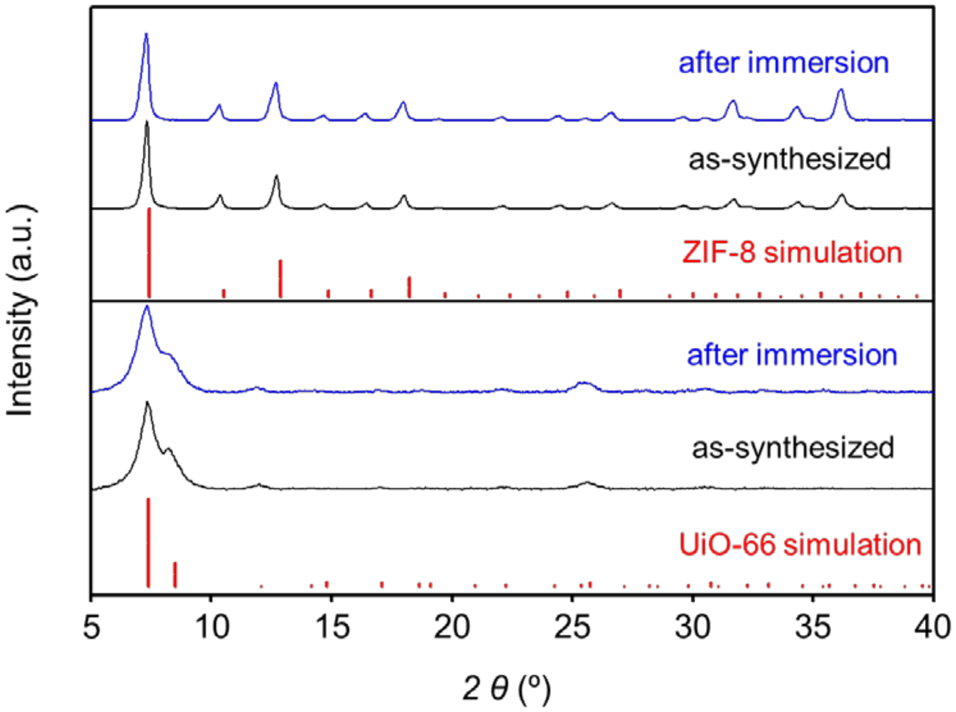

Supplementary Figure 16. PXRD analysis of BGL@UiO-66-NH_2 and BGL@ZIF-8 treated with neutral buffer.

PXRD analysis showing the stability of BGL@UiO-66-NH_2 and BGL@ZIF-8 before/after immersion in a Tris buffer (pH 7.0, 50 mM). Materials were immersed in the buffer for 30 min. Note that all of the patterns closely approximate their simulated peaks, reinforcing the stability of the materials under neutral conditions.

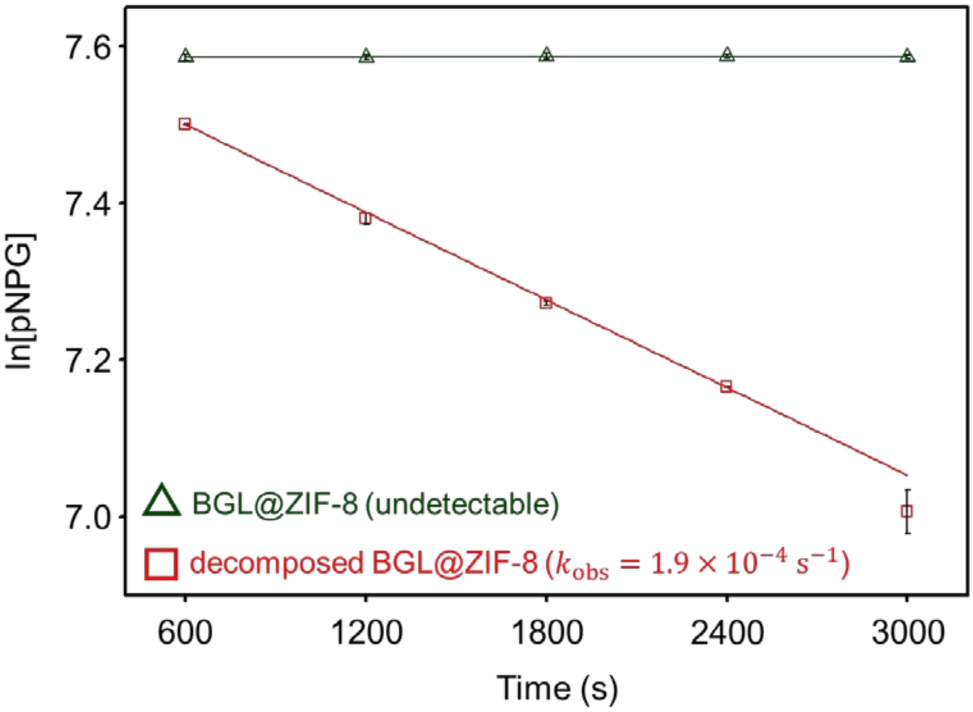

Supplementary Figure 17. The biological activity of BGL@ZIF-8.

Olive line: the activity of BGL@ZIF-8 with the ZIF-8 shell intact. Red line: the activity of BGL@ZIF-8 after the ZIF-8 shell has been decomposed, allowing the substrate to reach the BGL catalytic surface. Olive line: Error bars are standard deviations (n = 2). Red line: Error bars are standard deviations (n = 3). Source data are provided as a Source Data file.

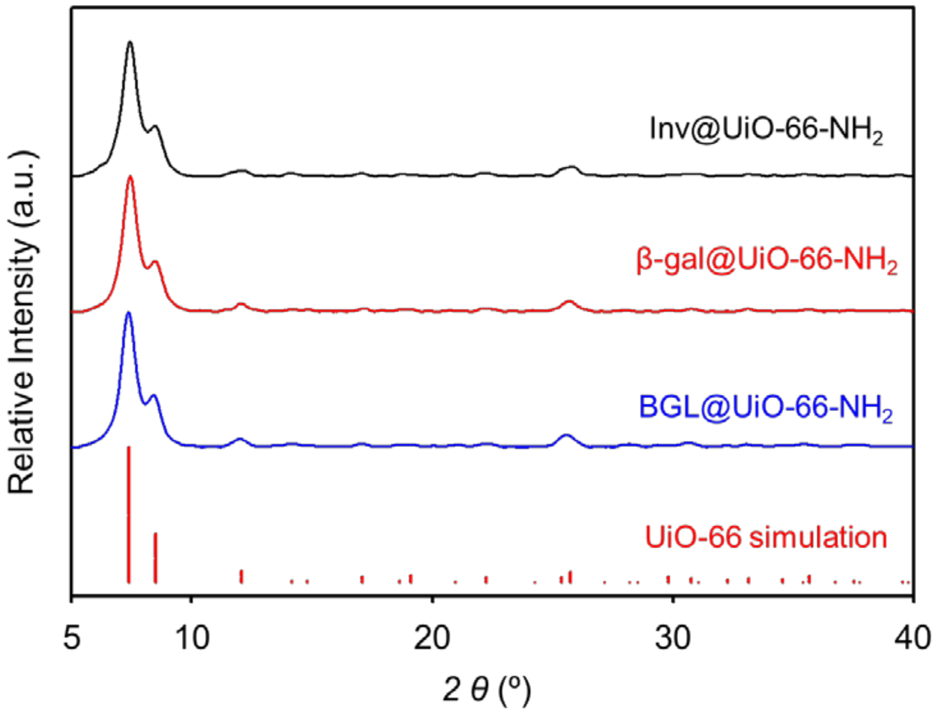

Supplementary Figure 18. PXRD analysis of glycosidases@UiO-66-NH_2.

PXRD analysis showing that the glycosidase@UiO-66-NH_2 samples display the characteristic peaks of UiO-66.

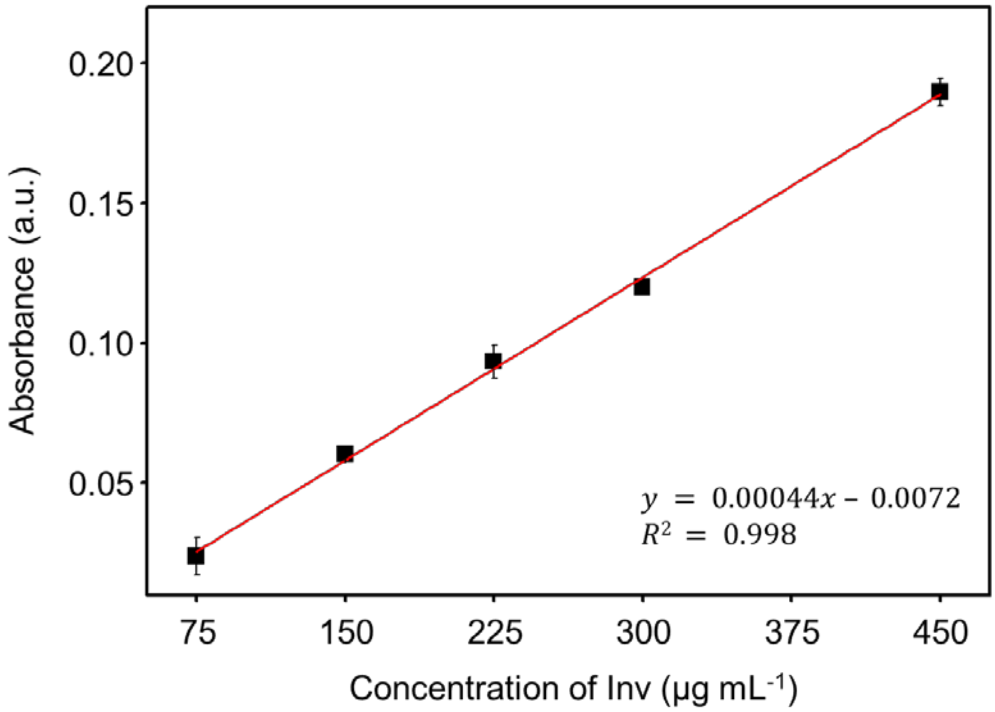

Supplementary Figure 19. Bradford assay of the Inv concentration.
The corresponding standard calibration line of the Bradford assay is shown above. Error bars are standard deviations (*n* = 3). Source data are provided as a Source Data file.

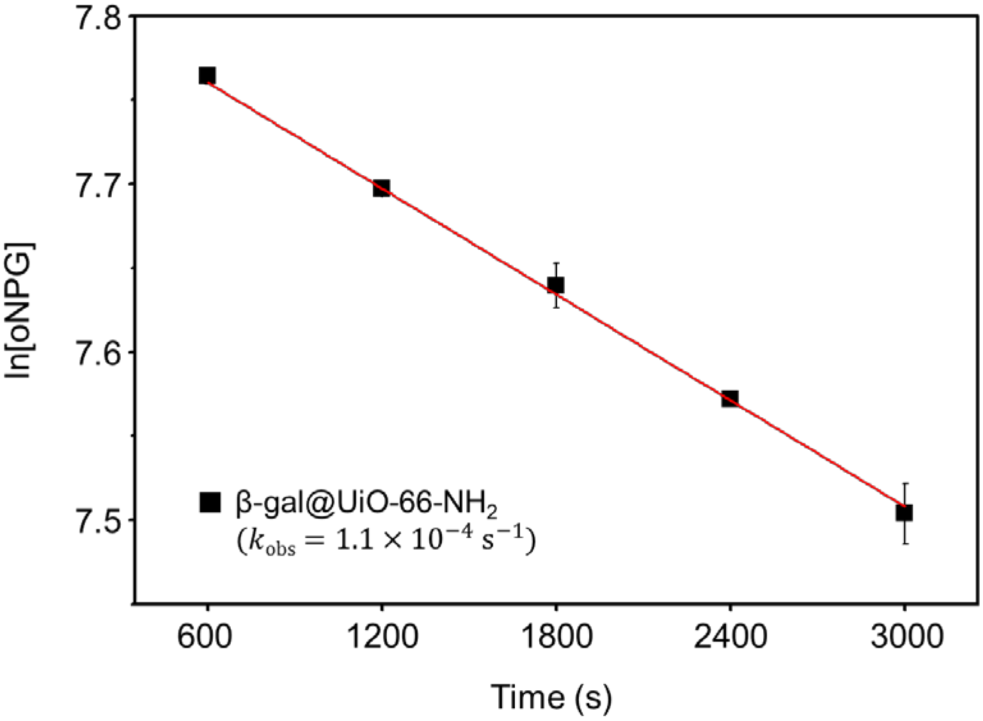

Supplementary Figure 20. The biological activity of β-gal@UiO-66-NH$_2$.
Error bars are standard deviations (*n* = 2). Source data are provided as a Source Data file.

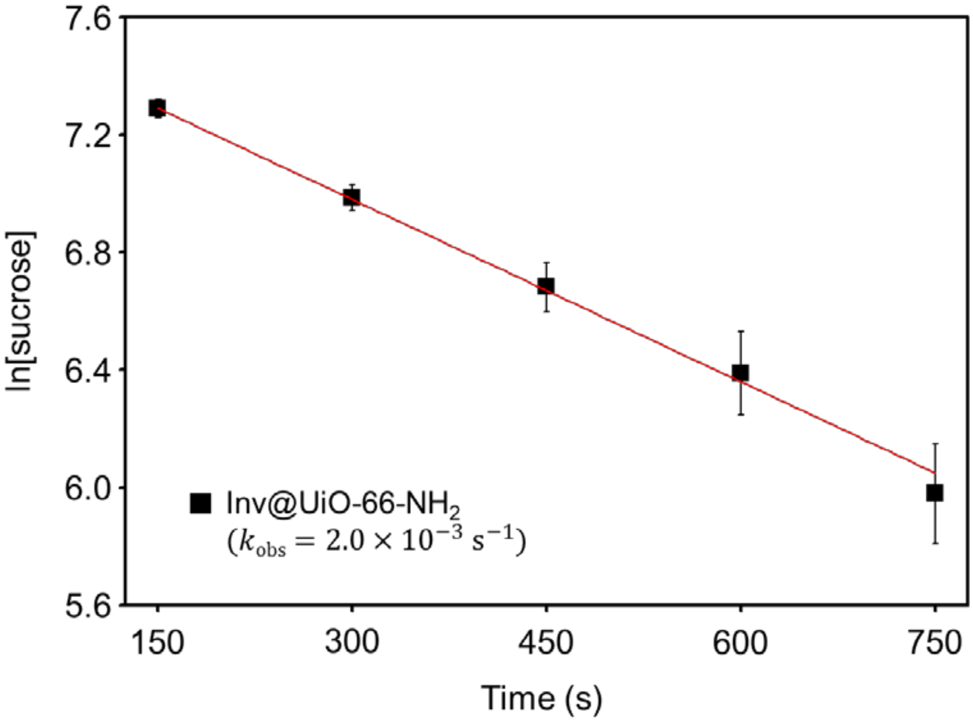

Supplementary Figure 21. The biological activity of Inv@UiO-66-NH$_2$.
Error bars are standard deviations (*n* = 3). Source data are provided as a Source Data file.

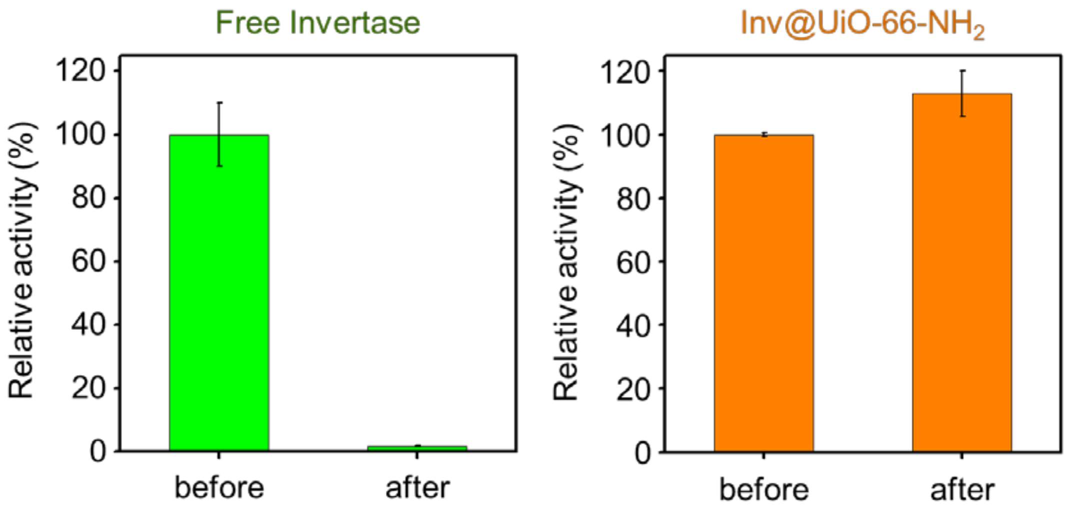

Supplementary Figure 22. The relative activity of samples incubated with protease. Free Inv (green bars) and Inv@UiO-66-NH_2 (orange bars) are shown above. Error bars are standard deviations (n = 3). Source data are provided as a Source Data file.

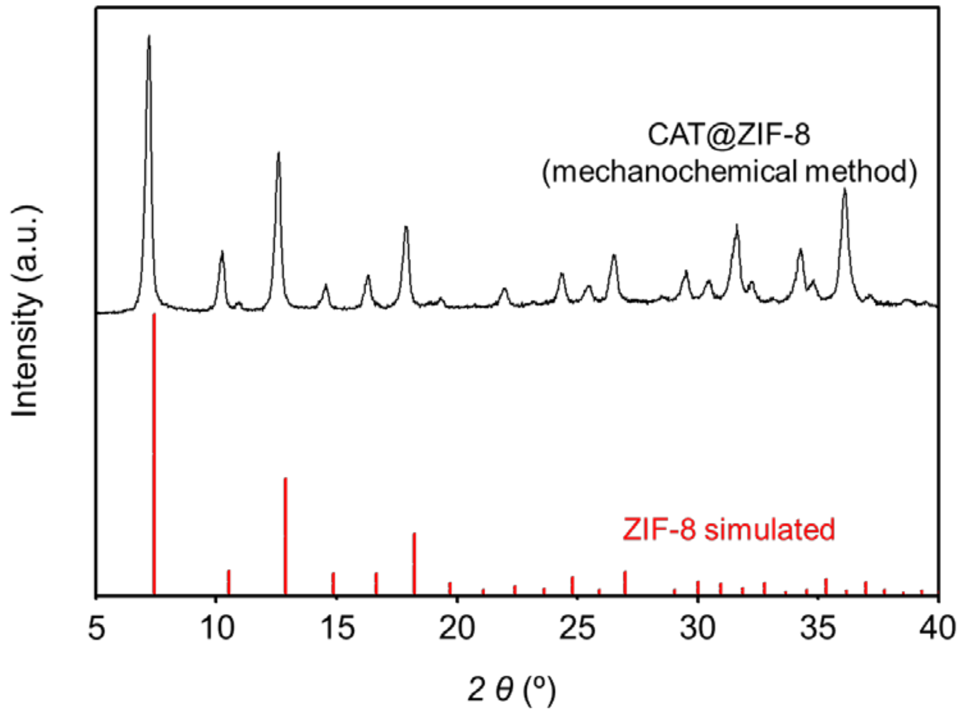

Supplementary Figure 23. PXRD analysis of CAT@ZIF-8.

PXRD analysis showing that CAT@ZIF-8 displays the characteristic peaks of ZIF-8 seen in the simulation.

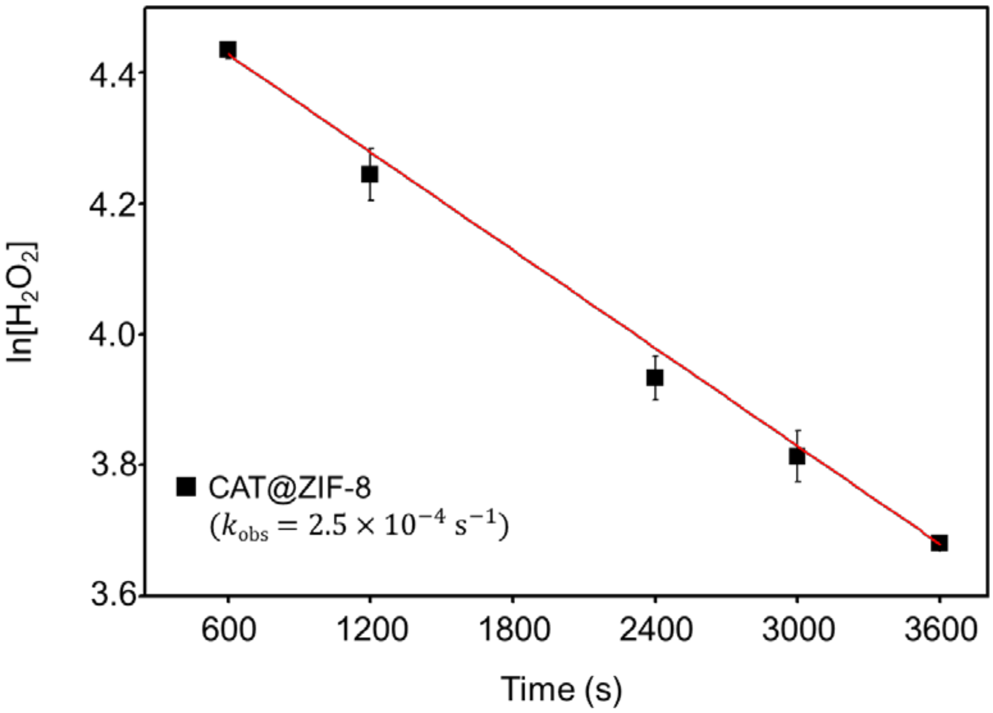

Supplementary Figure 24. The biological activity of CAT@ZIF-8.

Error bars are standard deviations (n = 3). Source data are provided as a Source Data file.

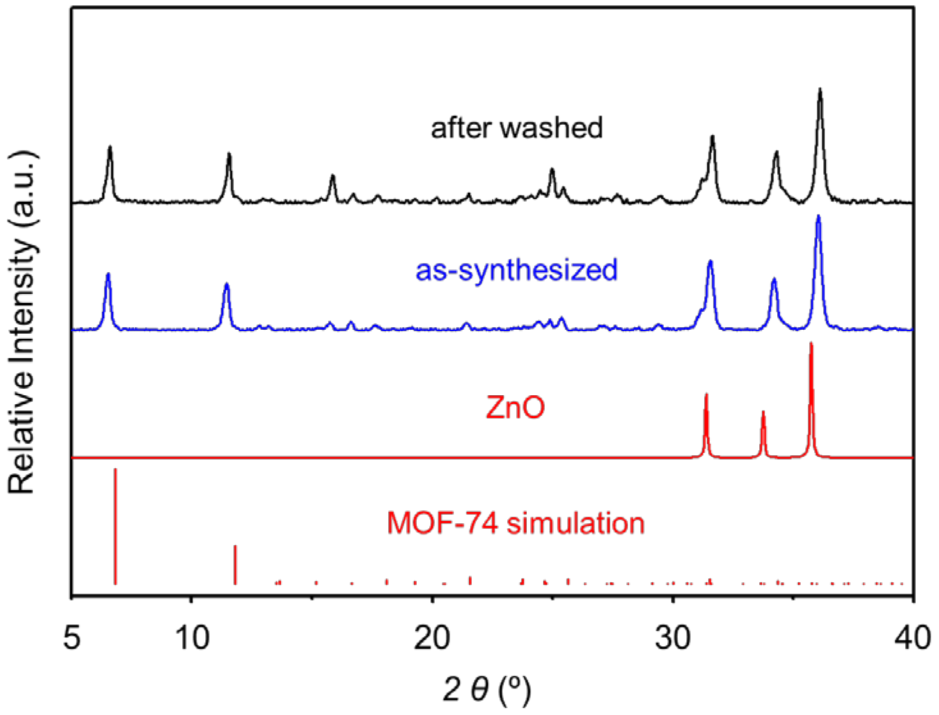

Supplementary Figure 25. PXRD analysis of CAT@Zn-MOF-74.

To show the stability of CAT@Zn-MOF-74 after immersion in water. The blue line is the PXRD result of as-synthesized CAT@Zn-MOF-74, obtained through the mechanochemical method. The black line is the PXRD result of CAT@Zn-MOF-74, obtained through the mechanochemical method and washed for 90 min. The red line is a characteristic zinc oxide spectrum. The red bar is a simulated spectrum characteristic of Zn-MOF-74.

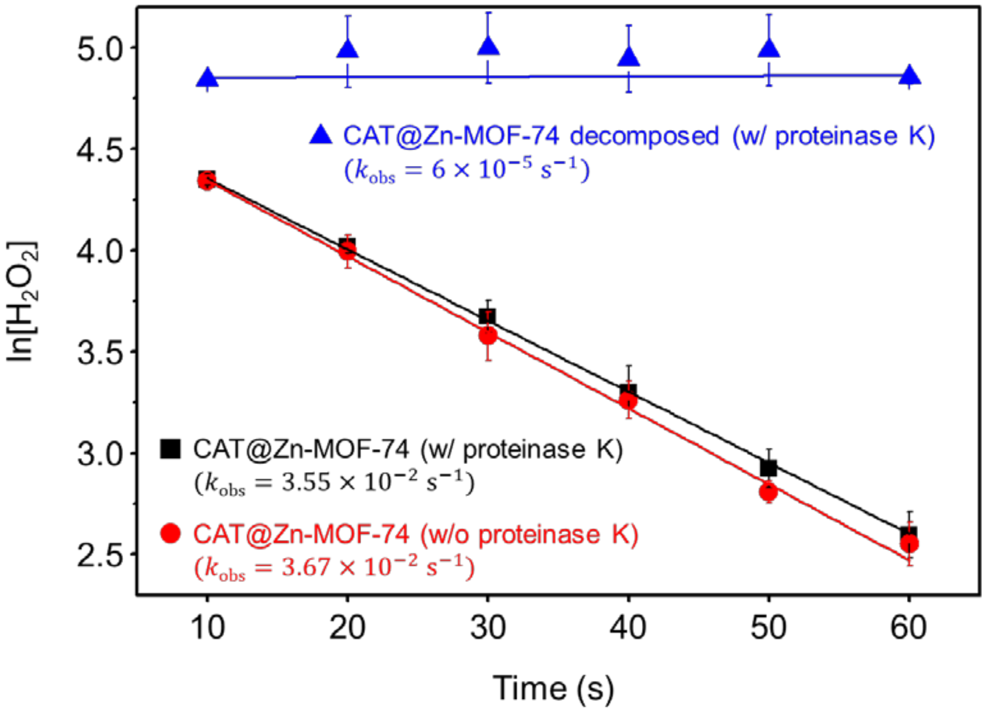

Supplementary Figure 26. The biological activity of CAT@Zn-MOF-74.
Blue triangles: the activity of CAT@Zn-MOF-74, which is decomposed by 1.875 M NaOH (pH~ 8.0) and then incubated with proteinase K for 30 min. Black squares: the activity of CAT@Zn-MOF-74 treated by proteinase K for 30 min. Red dots: the activity of CAT@Zn-MOF-74 without proteinase K treatment. Error bars are standard deviations (n = 3). Source data are provided as a Source Data file.

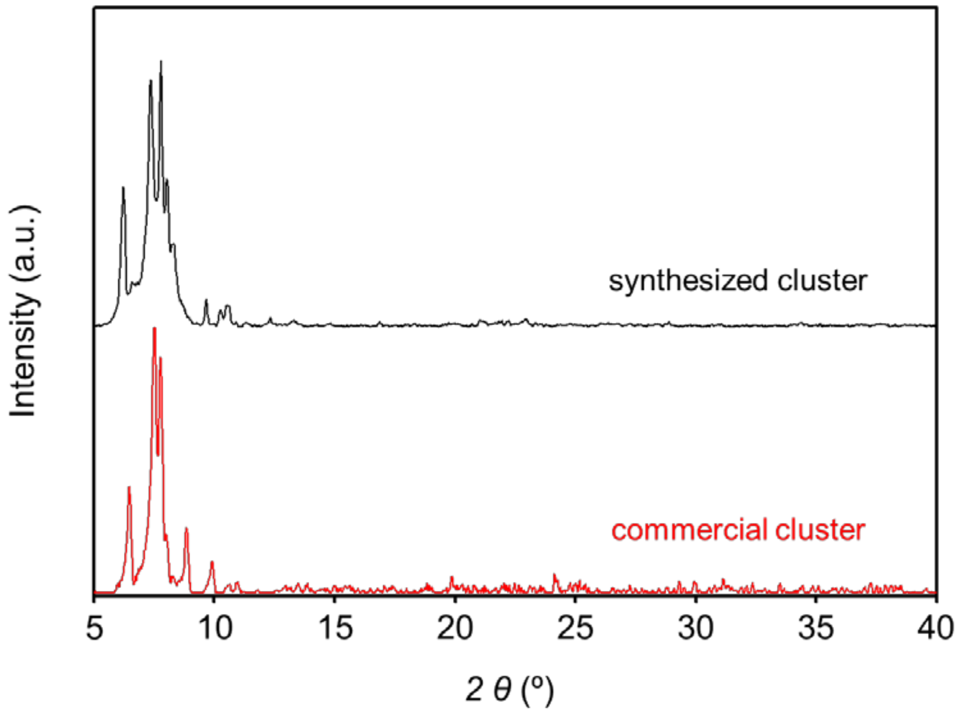

Supplementary Figure 27. PXRD analysis of MOF-precursor zirconium clusters. Commercial clusters were purchased from sigma and their PXRD spectrum is shown in red. Synthesized clusters were made following a published method[1] and their PXRD spectrum is shown in black.

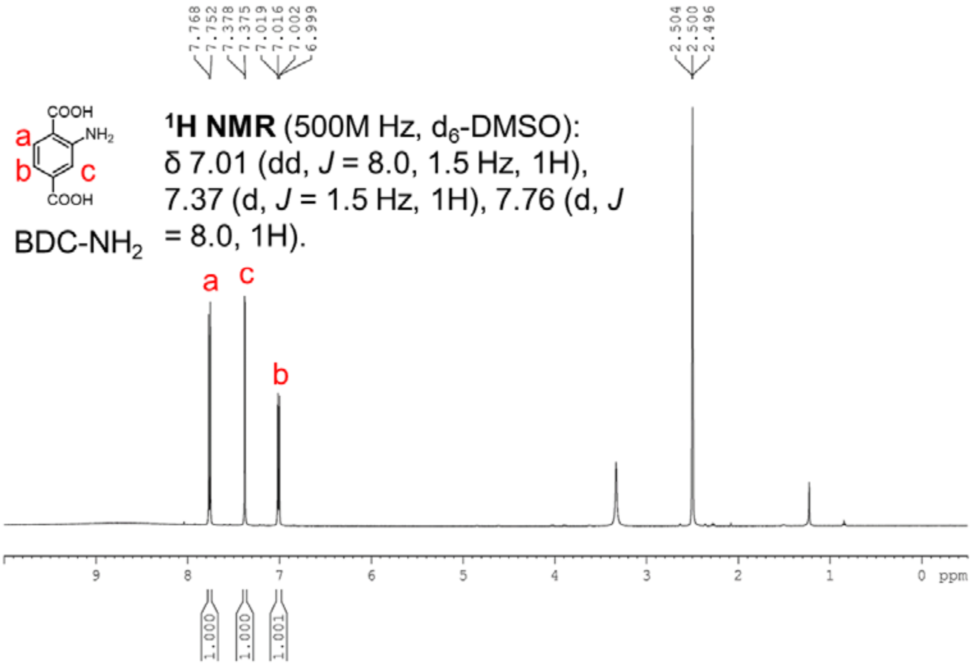

Supplementary Figure 28. ^{1}H NMR spectrum of BDC-NH_2.

^{1}H NMR (500 MHz, d_6-DMSO): δ 7.01 (dd, J = 8.0, 1.5 Hz, 1 H), 7.37 (d, J = 1.5 Hz, 1 H), 7.76 (d, J = 8.0, 1 H).

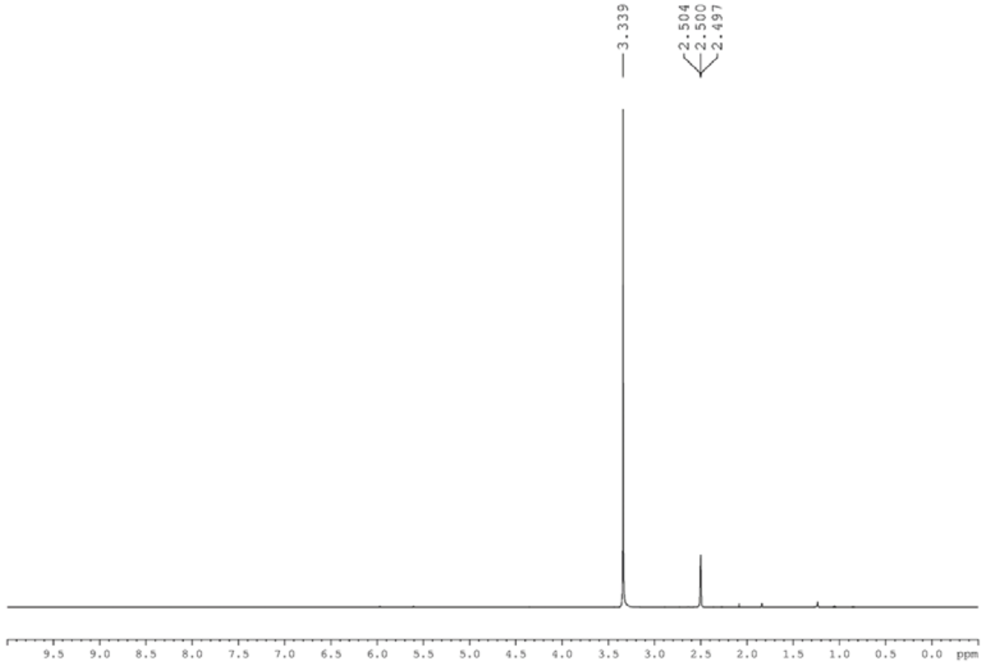

Supplementary Figure 29. ^{1}H NMR spectrum of the supernatant of BGL@UiO-66-NH_2 after washing.

No linker signal is detected confirming the removal of unreacted BDC-NH_2.

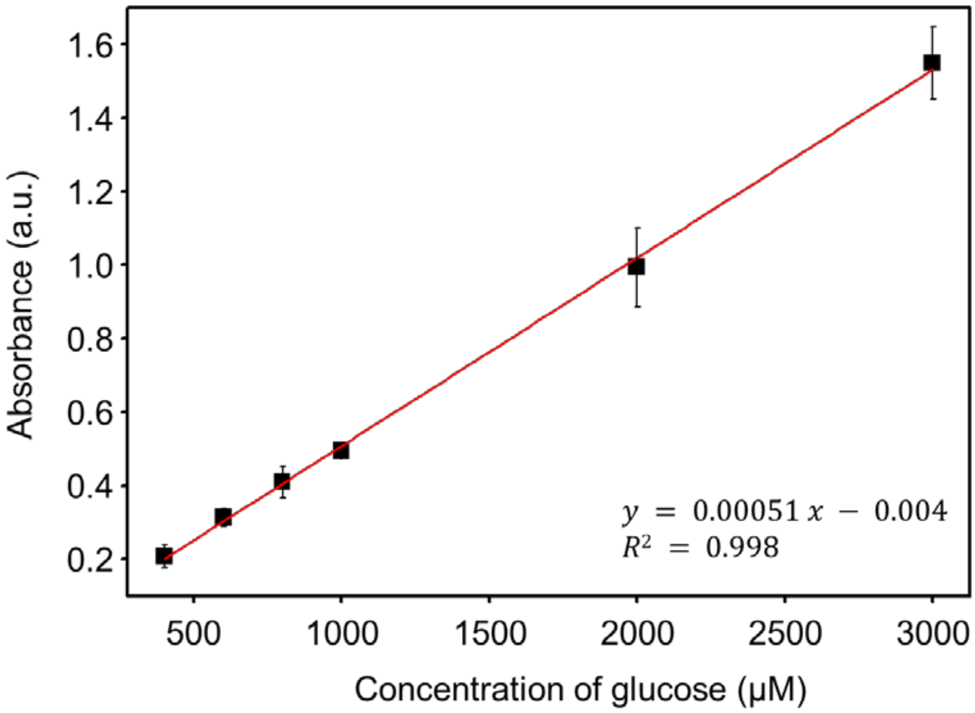

Supplementary Figure 30. Concentration of glucose.

The corresponding standard calibration line of the PAHBAH assay is shown above. Error bars are standard deviations (*n* = 3). Source data are provided as a Source Data file.

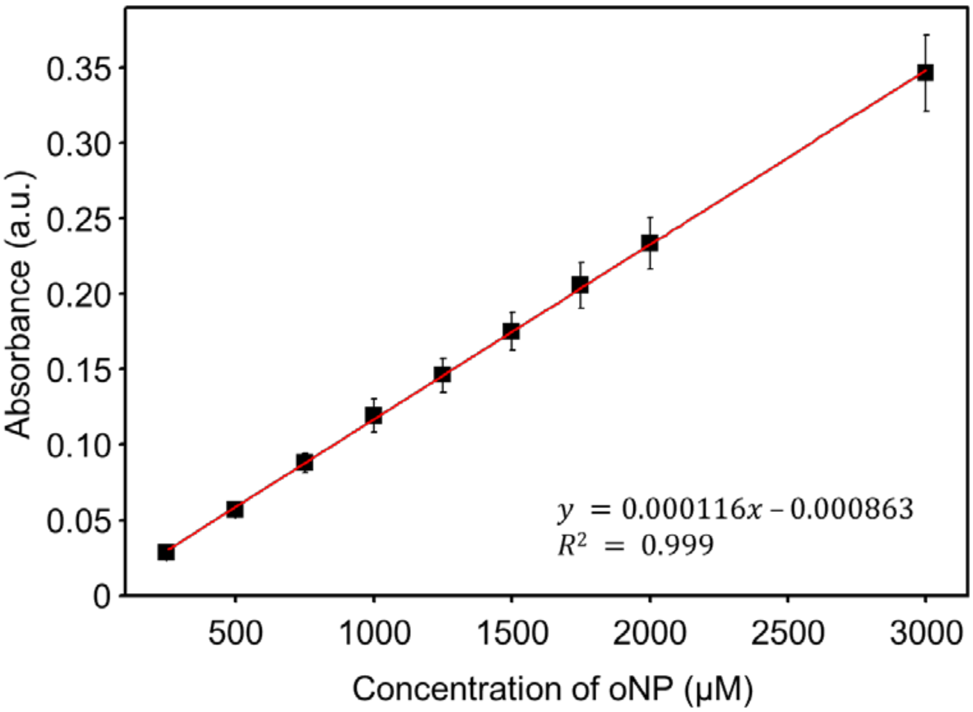

Supplementary Figure 31. Concentration of oNP.

The corresponding standard calibration line of the oNP is shown above. Error bars are standard deviations (n = 3). Source data are provided as a Source Data file.

Supplementary Tables

Supplementary Table 1: The kinetic parameters on β-glucosidase in BGL@UiO-66-NH_2

	K_M (mM)	V_{max} (μM·s^{-1})	k_{cat} (s^{-1})
**BGL@UiO-66-NH_2**	6.134	0.870	0.294

Supplementary References

1. Užarević K, *et al.* Mechanochemical and solvent-free assembly of zirconium-based metal–organic frameworks. *Chem. Commun.* **52**, 2133-2136 (2016).
